# A Transcriptome-Level Study Identifies Changing Expression Profiles for Ossification of the *Ligamentum Flavum* of the Spine

**DOI:** 10.1016/j.omtn.2018.07.018

**Published:** 2018-08-07

**Authors:** Yawei Han, Yuheng Hong, Liandong Li, Tengshuai Li, Zhen Zhang, Jingzhao Wang, Han Xia, Yutao Tang, Zhemin Shi, Xiaohui Han, Ting Chen, Qi Liu, Mengxia Zhang, Kun Zhang, Wei Hong, Yuan Xue

**Affiliations:** 1 Department of Histology and Embryology, School of Basic Medical Sciences, Tianjin Medical University, Tianjin, China; 2 School of Medical Imaging, Tianjin Medical University, Tianjin, China; 3 Department of Orthopaedics, Tianjin Medical University General Hospital, Tianjin, China

**Keywords:** non-coding RNA, ossification, *ligamentum flavum*, lncRNA, microRNA, circRNA, transcriptome

## Abstract

Ossification of the *ligamentum flavum* (OLF) is a common spinal disorder that causes myelopathy and radiculopathy. Non-coding RNAs (ncRNAs) are involved in numerous pathological processes; however, very few ncRNAs have been identified to be correlated with OLF. Here we compared the expression of lncRNA, mRNA, circRNA, and microRNA in OLF tissues from OLF patients and healthy volunteers through mRNA, lncRNA, and circRNA microarrays and microRNA sequencing. A total of 2,054 mRNAs, 2,567 lncRNAs, 627 circRNAs, and 28 microRNAs (miRNAs) were altered during the process of OLF. qPCR confirmed the differential expression of selected mRNAs and ncRNAs. An lncRNA-mRNA co-expression network, miRNA-mRNA target prediction network, and competing endogenous RNA (ceRNA) network of circRNA-miRNA-mRNA were constructed based on a correlation analysis of the differentially expressed RNA transcripts. Subsequently, gene ontology (GO) and Kyoto Encyclopedia of Genes and Genomes (KEGG) pathway analyses for the differentially expressed mRNAs and the predicted miRNAs target genes were performed. In addition, a deregulated miRNA-19b-3p-based miRNA-circRNA-lncRNA-mRNA network was confirmed, by gain-of-function and loss-of-function experiments, to function in the process of ossification. Taken together, this study provides a systematic perspective on the potential function of ncRNAs in the pathogenesis of OLF.

## Introduction

Ossification of the *ligamentum flavum* (OLF) of the spine is characterized by ectopic bone formation in the OLF and is highly prevalent in the population of East Asia.^1^, ^2^ The ossified ligaments form osteophytes that gradually increase in size, which, in turn, causes compression of the spinal cord and may lead to severe neurological symptoms. Many factors, such as genetic background, dietary habits, metabolic abnormalities, and mechanical stress, contribute to OLF.^3^, ^4^, ^5^ Additionally, many protein-coding genes, especially RUNX2, have been reported to be associated with the development of OLF and other ossification-related events, such as chondrocyte maturation, endochondral ossification, and fetal bone development.^6^, ^7^ However, the exact pathogenesis of OLF still remains unclear.

The number of human protein-coding genes is less than 2% of the whole genome sequence, whereas the vast majority of transcripts consist of non-coding RNAs (ncRNAs), including microRNA (miRNA), circular RNA (circRNA), and long ncRNA (lncRNA).^8^, ^9^ With multiple and diverse targets, ncRNAs are involved in numerous biological functions and pathological processes, including development, proliferation, apoptosis, survival, differentiation, and carcinogenesis.^10^, ^11^, ^12^, ^13^ The specific contribution of selected ncRNAs to osteogenic differentiation has been described.^14^ Recent studies reported that the process of osteogenic differentiation is governed by distinct miRNAs and lncRNAs. For instance, miR-214 has a crucial role in suppressing bone formation by inhibiting osteoblast activity through targeting ATF4.^11^ Moreover, it has been reported that bone mesenchymal stem cell (BMSC)-specific inhibition of miR-188 by intra–bone marrow injection of antagomiR-188 increases bone formation and decreases bone marrow fat accumulation in aged mice.^15^ Additional studies reported that H19 mediates BMP9-induced osteogenic differentiation of mesenchymal stem cells (MSCs) through Notch signaling^16^ and that MEG3 inhibits adipogenesis and promotes osteogenesis of MSCs via miR-140-5p.^17^ Although the field is developing, little is known about the functions of lncRNAs and circRNAs in OLF. Specifically, studies to date lack accurate lncRNA and circRNA profiling of OLF tissues.

In this study, we investigated the differentially expressed patterns of miRNA using miRNA sequencing and the differentially expressed patterns of lncRNA, mRNA, and circRNA using a microarray in OLF tissues. The randomly selected differential expression of representative miRNAs, lncRNAs, mRNAs, and circRNAs were further confirmed using qPCR. Additionally, we constructed a co-expression network of lncRNA-mRNA, a target prediction network of miRNA-mRNA, and a competing endogenous RNA (ceRNA) mechanism network of circRNA-miRNA-mRNA based on a correlation analysis between the differentially expressed RNA transcripts for the first time in OLF. Subsequently, the differentially expressed mRNAs and the predicted miRNAs target genes followed by gene ontology (GO) and Kyoto Encyclopedia of Genes and Genomes (KEGG) pathway analyses were performed. This study not only provides a systematic perspective on the potential function of ncRNAs and mRNAs during the process of OLF but also illuminates the potential mechanism of OLF pathogenesis and provides new targets for OLF.

## Results

### Identification of Differentially Expressed mRNAs in OLF

**Figure 1:**
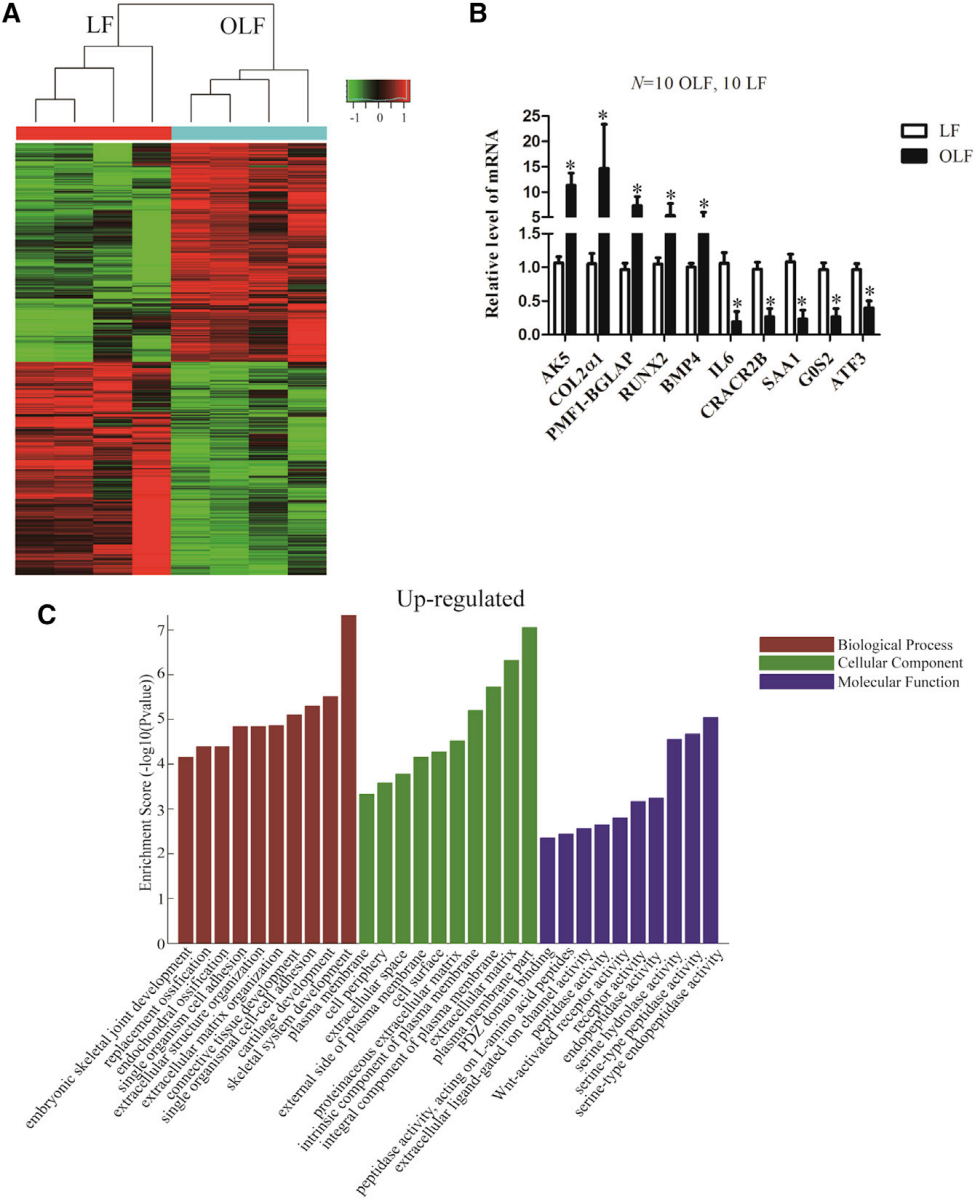
Expression Profile of mRNAs in OLF and Normal LF (A) Microarray analysis for mRNA was performed with RNA extracted from ossified (n = 4) and normal (n = 4) *ligamentum flavum*. Hierarchical cluster analysis of significantly differentially expressed mRNAs: bright green, under-expression; gray, no change; bright red, overexpression. (B) Ten differentially expressed representative mRNAs were validated in OLF and normal LF tissues by qPCR (n = 10 per group). GAPDH was used as an internal control. (C) GO annotation of the linear counterparts of upregulated mRNAs with the top ten enrichment scores covering domains of biological processes, CCs, and MFs was performed. Data are presented as mean ± SEM; p values were analyzed by Student’s t test; *p < 0.05.

First, we collected ossified and normal LF tissues from patients during surgery; information about the patients and the section of ossification are shown in Table S1. The mRNA expression profiles were detected in 4 OLF and 4 normal LF tissues, and hierarchical clustering (Figure 1A) was performed to show distinguishable mRNA expression patterns among samples. From the mRNA expression profiling data, 2,054 mRNAs were differentially expressed in OLF tissues (fold change > 2, p < 0.05), of which 1,041 mRNAs were upregulated, and 1,013 mRNAs were downregulated. Five upregulated and five downregulated mRNAs were randomly selected to verify the microarray data in extension samples of OLF and normal LF tissues by qRT-PCR, and the results were consistent with the microarray data (Figure 1B; Table S2).

Subsequently, we conducted the GO and KEGG pathway analyses, and the results showed that the top ten most enriched GO terms of the upregulated genes include endochondral ossification, replacement ossification, extracellular matrix, and Wnt-activated receptor activity (Figure 1C), which are associated with ossification and osteogenic differentiation. The top ten most enriched GO terms of the downregulated genes are shown in Figure S1A. KEGG pathway analysis revealed that the pathways of upregulated genes (Figure S1B) and downregulated genes (Figure S1C) were associated with osteogenic differentiation, including extracellular matrix (ECM) receptor interaction, nuclear factor κB (NF-κB) signaling, tumor necrosis factor (TNF) signaling, osteoclast differentiation, mammalian target of rapamycin (mTOR) signaling, and mitogen-activated protein kinase (MAPK) signaling. Thus, we identified subsets of mRNAs that are differentially regulated during the process of OLF by applying a systematic array approach.

### Identification of Differentially Expressed lncRNAs in OLF

**Figure 2:**
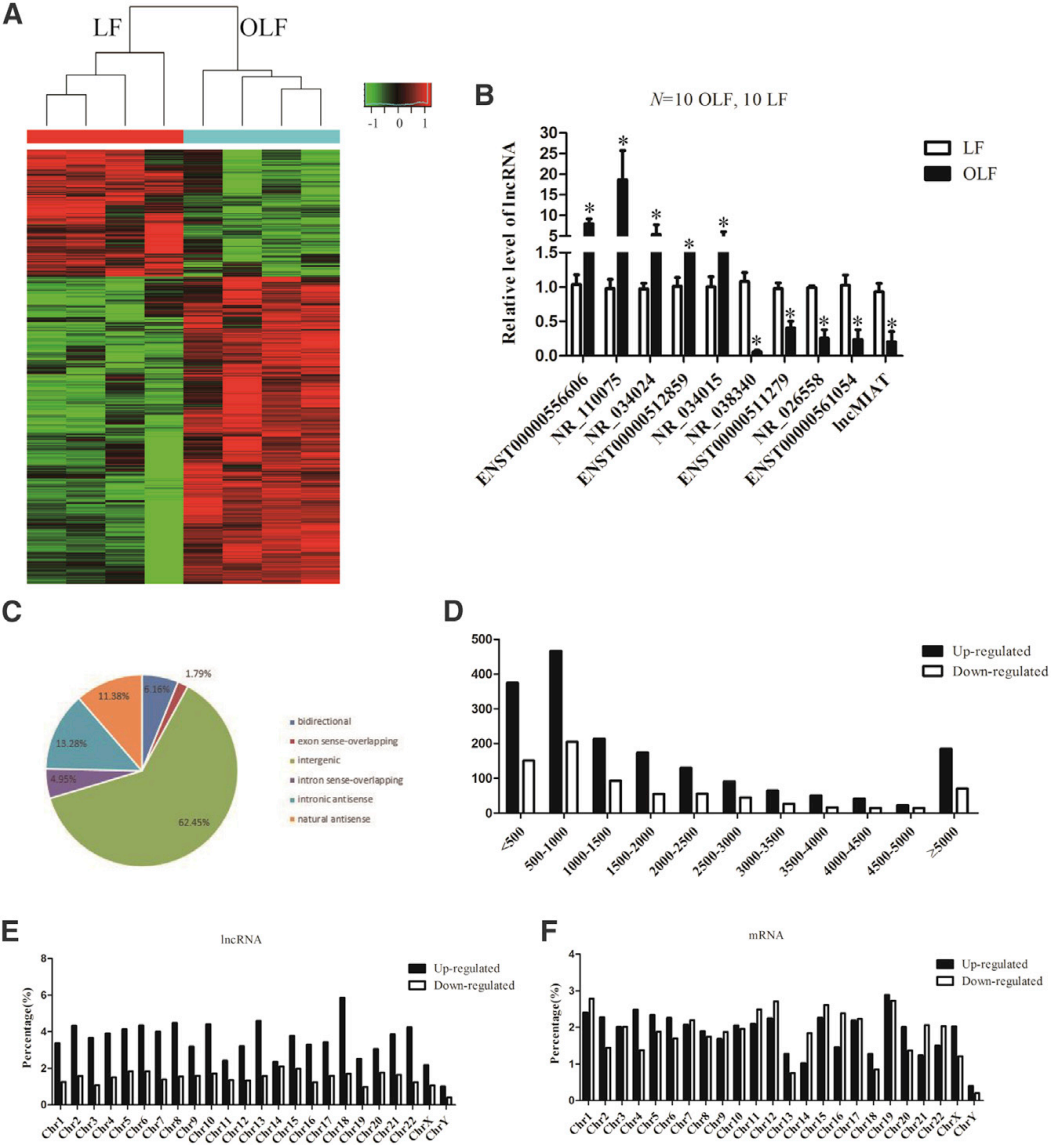
The Differentially Expressed lncRNAs in OLF (A) Microarray analysis for lncRNAs was performed with RNA extracted from ossification (n = 4) and normal (n = 4) *ligamentum flavum*. Hierarchical cluster analysis of significantly differentially expressed lncRNAs: bright green, under-expression; gray, no change; bright red, overexpression. (B) Differential expression of ten representative lncRNAs was validated in human ossified and normal *ligamentum flavum* tissues by qPCR (n = 10 per group). GAPDH was used as an internal control. (C) The deregulated lncRNAs were divided into intergenic, antisense, natural antisense, bidirectional, intron sense-overlapping, and exon sense-overlapping. (D) The deregulated lncRNAs were classified according to different lengths. (E and F) The deregulated lncRNAs (E) and mRNAs (F) were classified according to their percentages in different chromosomes. Data are presented as mean ± SEM; p values were analyzed by Student’s t test; *p < 0.05.

To investigate the differentially expressed lncRNAs during the process of OLF, we performed lncRNA expression profiles in 4 OLF and 4 normal LF tissues, and hierarchical clustering (Figure 2A) showed distinguishable lncRNA expression patterns among samples. From the lncRNA expression profiling data, 2,567 lncRNAs were found to be differentially expressed in OLF tissues (fold change > 2, p < 0.05), of which 1,817 lncRNAs were upregulated and 750 lncRNAs were downregulated. The number of upregulated lncRNAs is much higher than that of the downregulated ones, suggesting that this is like a ligament-to-bone conversion. Subsequently, we randomly selected five upregulated and five downregulated lncRNAs to perform qRT-PCR to verify the microarray results in extension samples of OLF tissues and normal LF tissues. The results showed that the deregulated lncRNAs are consistent with the microarray data (Figure 2B; Table S3). Further lncRNAs subgroup analysis showed that the majority of differentially expressed lncRNAs in this study were intergenic (62.45%), and others include intronic antisense (13.28%), natural antisense (11.38%), bidirectional (6.16%), intron sense-overlapping (4.95%), and exon sense-overlapping (1.79%) (Figure 2C). Character analysis showed that the differentially expressed lncRNAs are mainly between 200 and 1,000 bp in length (Figure 2D). It has been reported that chromosomal imbalances are associated with various diseases.^18^ In this study, aberrantly expressed lncRNAs and mRNAs were found to be most prone to be located on chromosomes 18 and 19, respectively, after normalization with the amount of genes located on each chromosome (Figures 2E and 2F). Thus, we identified a series of lncRNAs that are differentially expressed between OLF and normal LF tissues.

**Figure 3:**
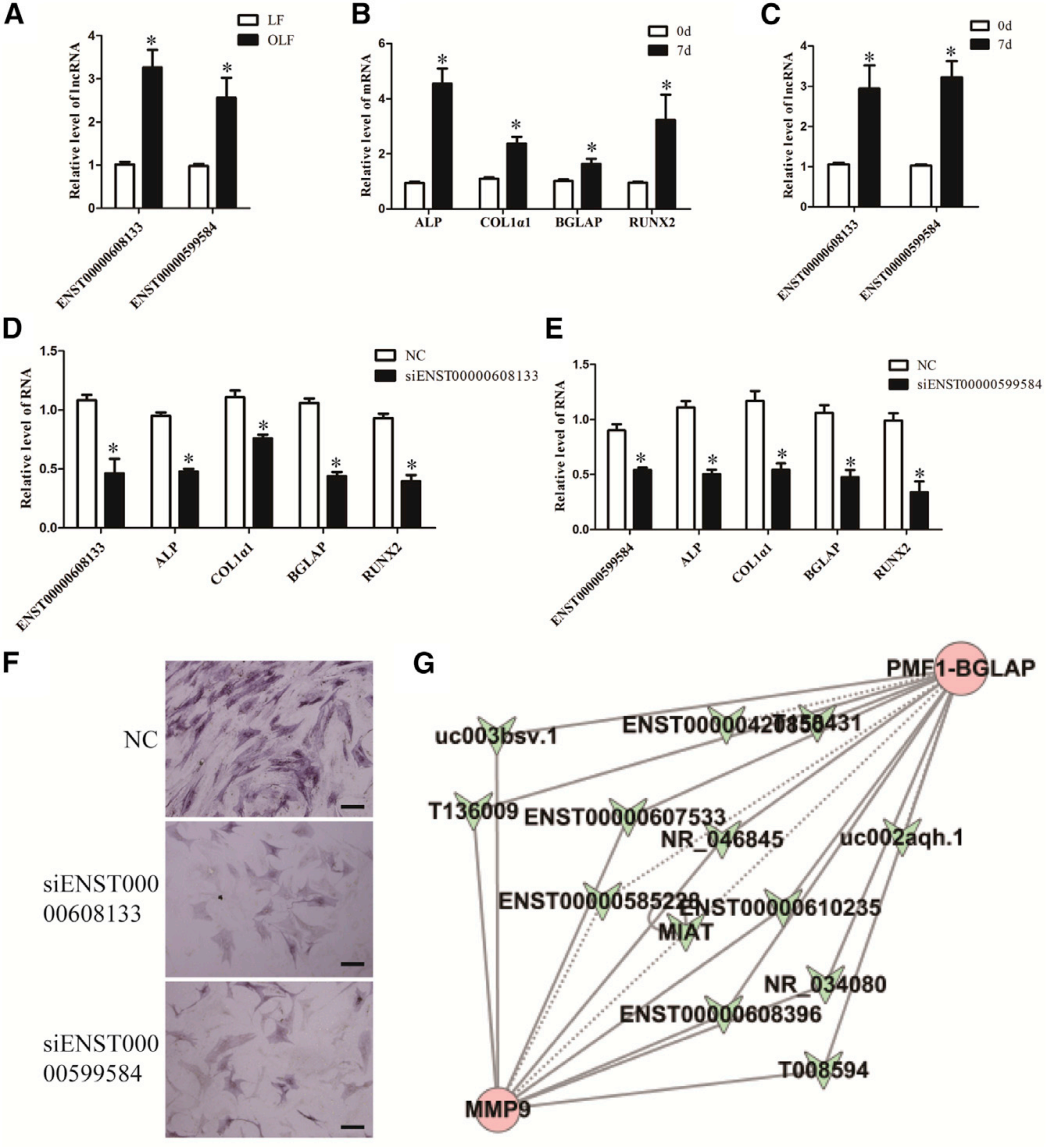
The Function of Two Upregulated lncRNAs and Co-expression Network of MMP9 and BGLAP with Their Associated lncRNAs (A) The differential expression of ENST00000608133 and ENST00000599584 was validated with total RNA isolated from OLF and normal LF by qPCR (n = 10 per group). GAPDH was used as an internal control. (B and C) qPCR analysis of ALP, COL1α1, BGLAP, and RUNX2 (B) and ENST00000608133 and ENST00000599584 (C) was performed, with total RNA isolated from hMSCs and treated with osteogenic medium for 0 and 7 days. (D and E) hMSCs were transfected with siENST00000608133 (D), siENST00000599584 (E), or the negative control and further cultured in osteogenic medium for 7 days. The RNA levels of ENST00000608133 (D), ENST00000599584 (E) ALP, Col1α1, BGLAP, and RUNX2 were detected by qPCR. (F) Representative images of ALP staining of hMSCs after transfection with siENST00000608133, siENST00000599584, or the negative controls and further culture in osteogenic medium for 7 days. Scale bars, 400 μm. (G) Co-expression network of MMP9 and BGLAP with their associated lncRNAs. The network is based on PCC. V nodes represent lncRNA, and circle nodes represent mRNAs. Solid lines represent a positive relationship, and dashed lines represent a negative relationship. Data are presented as mean ± SEM for at least triplicate experiments. The p values were analyzed by Student’s t test; *p < 0.05.

To further confirm that the deregulated lncRNAs function in the process of ossification, we selected two upregulated lncRNAs, ENST00000608133 and ENST00000599584, in OLF (Table S3). The transcripts of these two lncRNAs were upregulated in OLF and in human bone marrow mesenchymal stem cells (hMSCs) under osteogenic induction (Figures 3A–3C). Knockdown of these lncRNAs with the respective small interfering RNA (siRNA) reduced the mRNA levels of alkaline phosphatase (ALP), COL1α1, BGLAP, and RUNX2, all of which are osteogenic differentiation-associated genes, and ALP staining during the process of hMSC differentiation into osteoblasts (Figures 3D–3F). These data indicate that the deregulated lncRNAs can regulate ossification-associated genes.

### Construction of the lncRNA-mRNA Co-expression Network

To date, the functions of most lncRNAs have not been annotated, and the functional forecast of lncRNAs is made according to the annotations of the co-expressed mRNA function. Therefore, we selected ten differentially expressed mRNAs (RUNX2, MMP9, BGLAP, IL6, COL2α1, AKT1, TNFRSF1A, TGFB2, BMP4, and PIK3R3) that have been reported to be closely associated with ossification and osteogenic differentiation^7^, ^19^, ^20^, ^21^, ^22^, ^23^, ^24^, ^25^, ^26^ together with co-expressed lncRNAs in the microarray based on the degree of correlation to build a coding-non-coding (CNC) network to predict the functions of the deregulated lncRNAs (Figure S2). Our data showed that the co-expression network was composed of 704 network nodes and 748 connections among ten coding genes and lncRNAs. The co-expression network could imply that these lncRNAs are involved in the process of OLF. For instance, the sub-network of MMP9 and BGLAP showed that the downregulated lncRNA MIAT was negatively correlated with these mRNAs (Figure 3G), which is consistent with a study showing that the lncRNA MIAT inhibits osteogenic differentiation.^27^ Therefore, the CNC network could predict the functions of co-expressed lncRNAs in OLF.

### Identification of Differentially Expressed miRNAs in OLF

**Figure 4:**
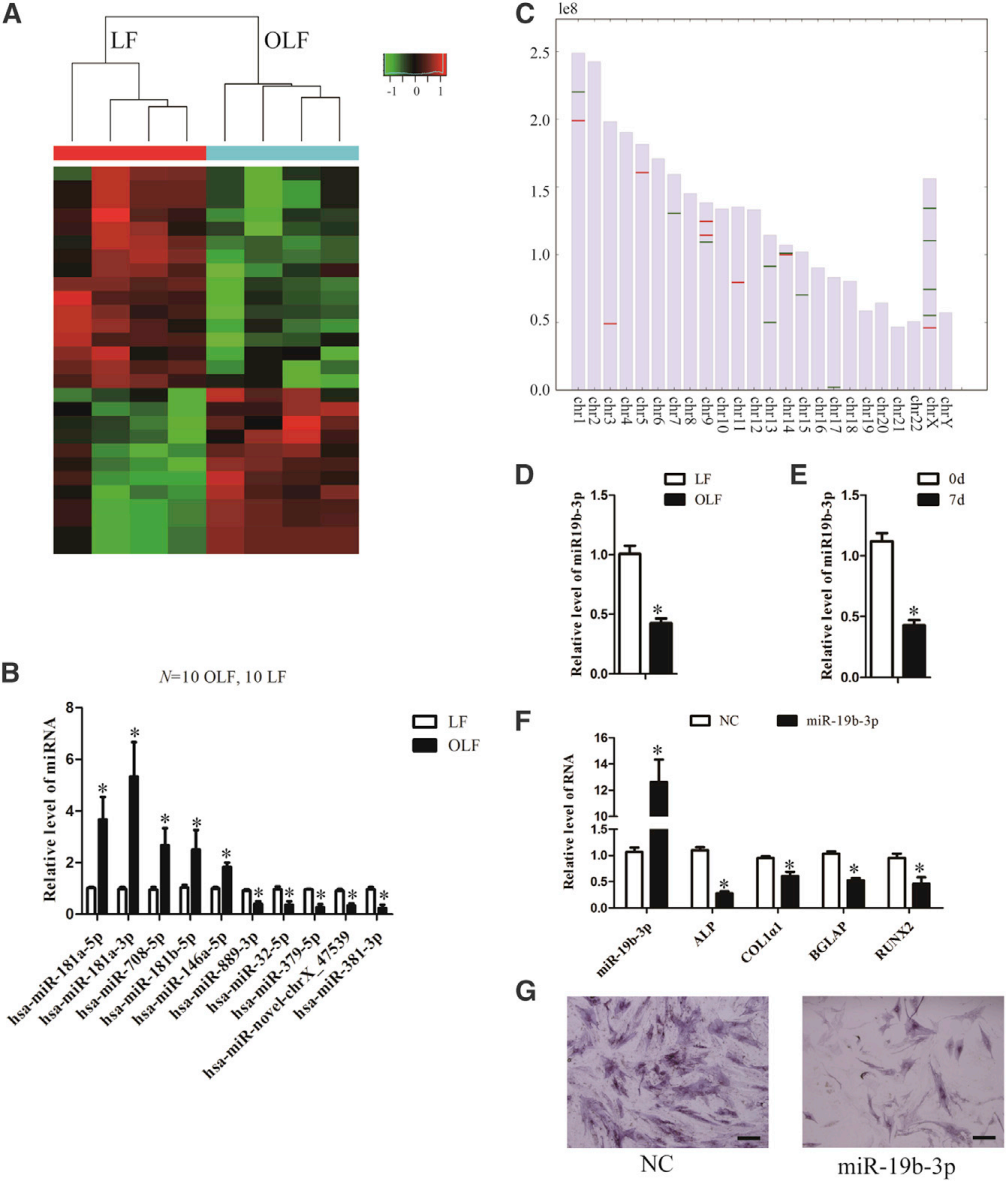
Differentially Expressed miRNAs in OLF and the Function of miR-19b-3p (A) Sequencing analysis for miRNA was performed with RNA extracted from ossification (n = 4) and normal (n = 4) LF. Hierarchical cluster analysis of significantly differentially expressed lncRNAs: bright green, under-expression; gray, no change; bright red, overexpression. (B) Differential expression of ten representative miRNAs was validated in human ossified and normal LF tissues by qPCR (n = 10 per group). (C) The differentially expressed miRNAs were distributed on different chromosomes. (D and E) qPCR analysis of miR-19b-3p was performed with total RNA isolated from ossified and normal LF (D) and hMSCs treated with osteogenic medium for 0 and 7 days (E). (F) hMSCs were transfected with miR-19b-3p mimic or the negative control and further cultured in osteogenic medium for 7 days. The RNA levels of miR-19b-3p, ALP, Col1α1, BGLAP, and RUNX2 were detected by qPCR. U6 was used as an internal control for miR-19b-3p. GAPDH was used as an internal control for mRNA. (G) Representative images of ALP staining of hMSCs after transfection with miR-19b-3p mimic or the negative controls and further culture in osteogenic medium for 7 days. Scale bars, 400 μm. Data are presented as mean ± SEM for at least triplicate experiments. The p values were analyzed by Student’s t test; *p < 0.05.

To identify the miRNA expression profiles in OLF, we performed miRNA sequencing, and the results showed that 12 upregulated and 16 downregulated miRNAs are identified in OLF (fold change > 2, p < 0.05) (Figure 4A). Subsequently, qPCR confirmed differential expression of these miRNAs between OLF and LF tissues (Figure 4B; Table S4). Moreover, the differentially expressed miRNAs were found to be prone to be located on chromosomes 1, 9, 14, and X (Figure 4C).

To further confirm the function of the deregulated miRNAs, we selected a downregulated miRNA, miR-19b-3p, from miRNA sequencing (Table S4). miR-19b-3p was downregulated in OLF and in hMSCs under osteogenic induction (Figures 4D and 4E). Overexpression of miR-19b-3p with mimics reduced the mRNA levels of the osteogenic differentiation-associated genes and ALP staining during the process of osteogenic differentiation (Figures 4F and 4G). These results indicate that miR-19b-3p regulates ossification-related genes.

### Construction of the Target Prediction Network of miRNA-mRNA

**Figure 5:**
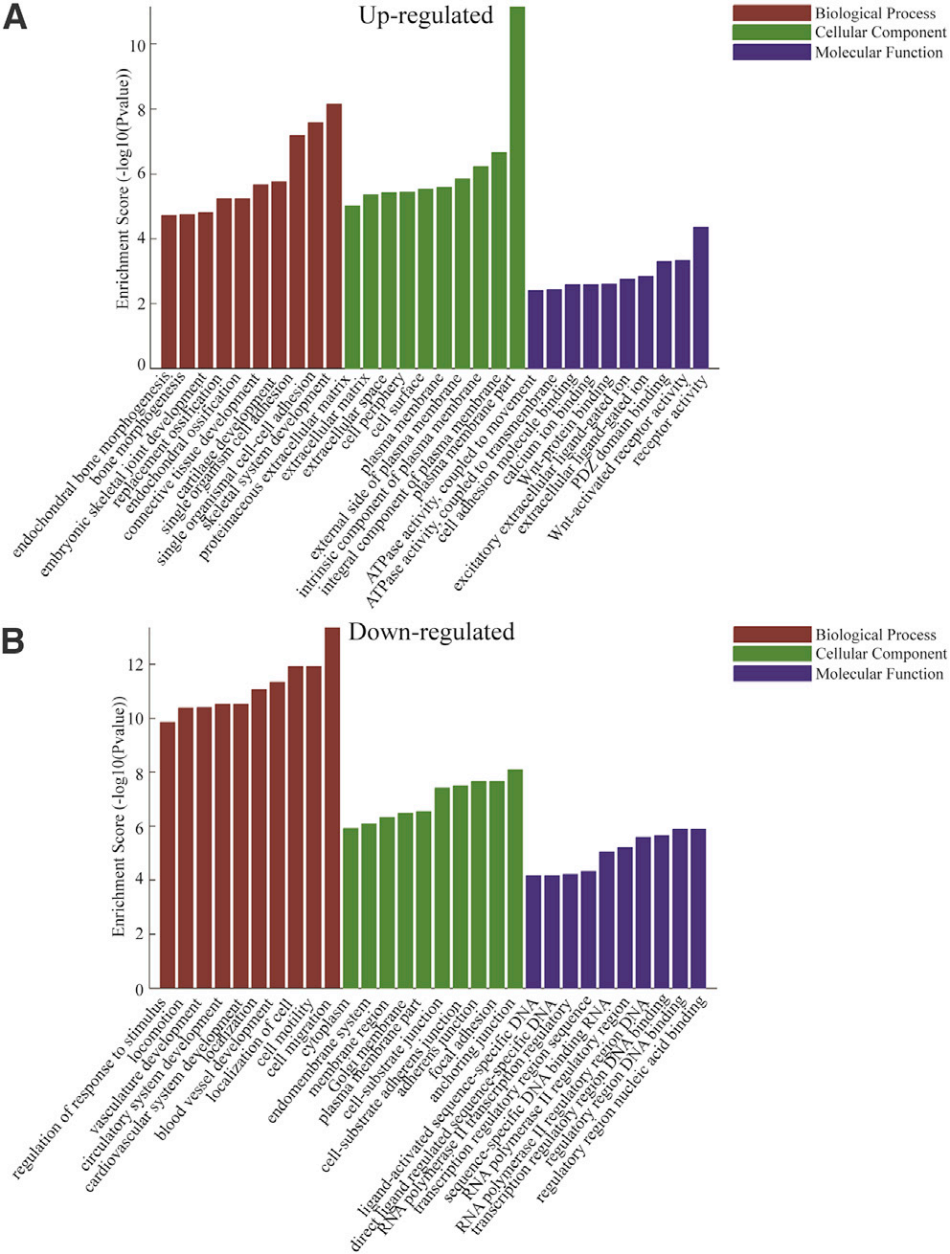
GO Analysis of the MicroRNA-mRNA Network (A and B) GO annotation of the linear counterparts of upregulated (A) and downregulated (B) mRNAs with the top ten enrichment score covering domains of biological processes, cellular components, and molecular functions was performed. Both up- and downregulated mRNAs are significantly changed, with fold change > 2.0, p < 0.05 between OLF and normal LF tissues.

**Figure 6:**
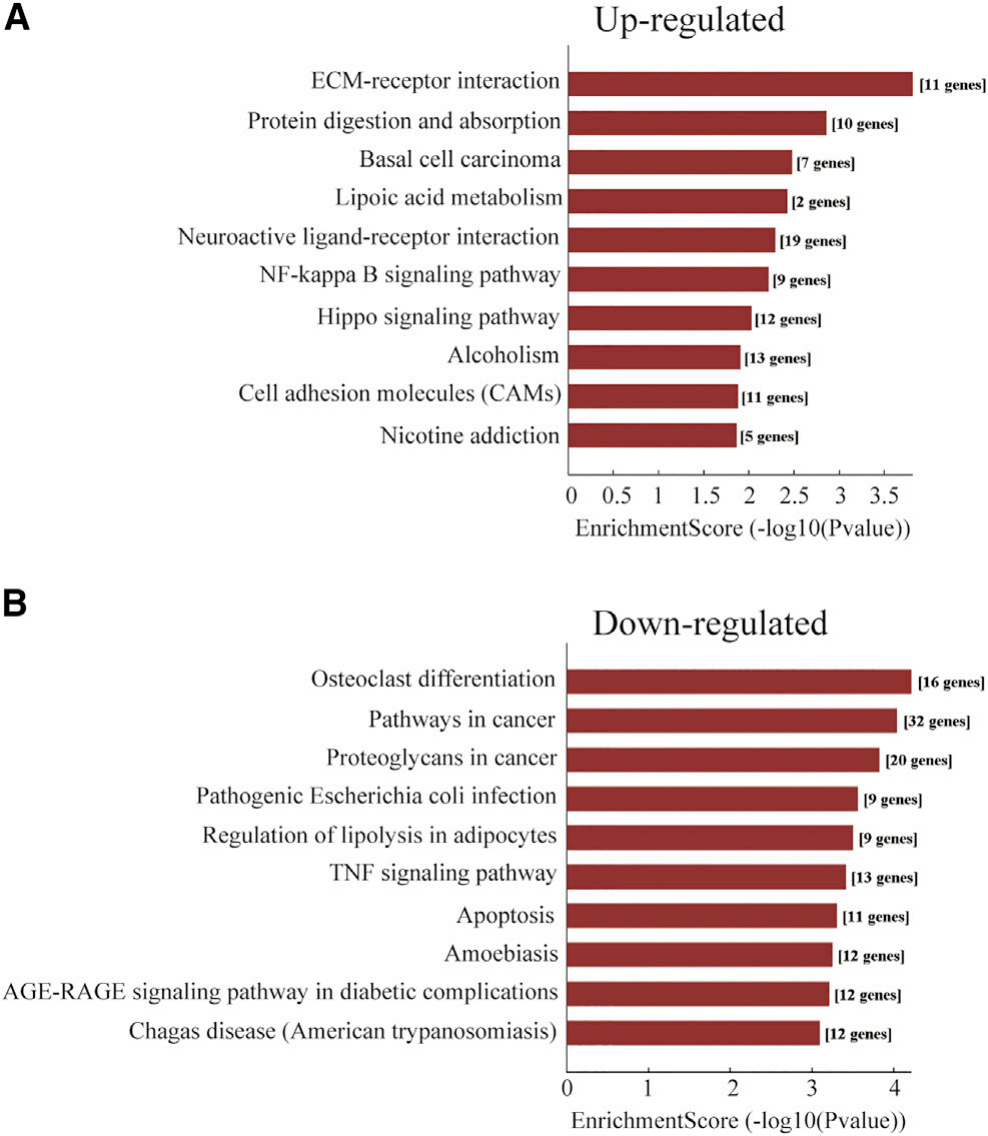
KEGG Analysis of the MicroRNA-mRNA network. (A and B) KEGG pathway enrichment analysis of upregulated (A) and downregulated (B) mRNAs with the top ten enrichment scores was performed. Both up- and downregulated mRNAs are significantly changed, with fold change > 2.0, p < 0.05 between OLF and normal LF tissues.

To identify the function of the aberrantly expressed miRNAs, the prediction of miRNA target genes was performed by the TargetScan, miRBase, and miRanda databases, and subsequently overlapped these databases. All of these predicted 1,201 mRNAs were used to perform the GO and KEGG pathway analysis (Figure S3A). However, the top ten signaling pathways, including the transforming growth factor β (TGF-β) signaling pathway, Wnt signaling pathway, and Hippo signaling pathway, are obviously associated with ossification (Figure S3B). To find a much more specific miRNA-mRNA interaction network, we used the differentially expressed mRNAs identified previously (Figure 1A) to overlap with the 1,201 predicted targets, and only genes that negatively correlated with their corresponding miRNAs were included (Figure S4). As shown in Figures 5A and 5B, the GO analysis showed that the differentially expressed miRNAs targets were mainly associated with skeletal system development, ECM, endochondral ossification, replacement ossification, and Wnt-activated receptor activity. Furthermore, KEGG pathway analysis also revealed that the upregulated mRNAs are associated with the signaling pathways of ossification and the downregulated mRNAs are associated with osteoclast differentiation, indicating that differentiation to osteoclasts is repressed in the process of ossification (Figures 6A and 6B). Taken together, we have identified subsets of miRNAs that are differentially regulated in OLF, and the miRNA-mRNA network implies that these miRNAs play a core role in the transcription-regulatory network during the process of OLF.

### Identification of Differentially Expressed circRNAs in OLF

**Figure 7:**
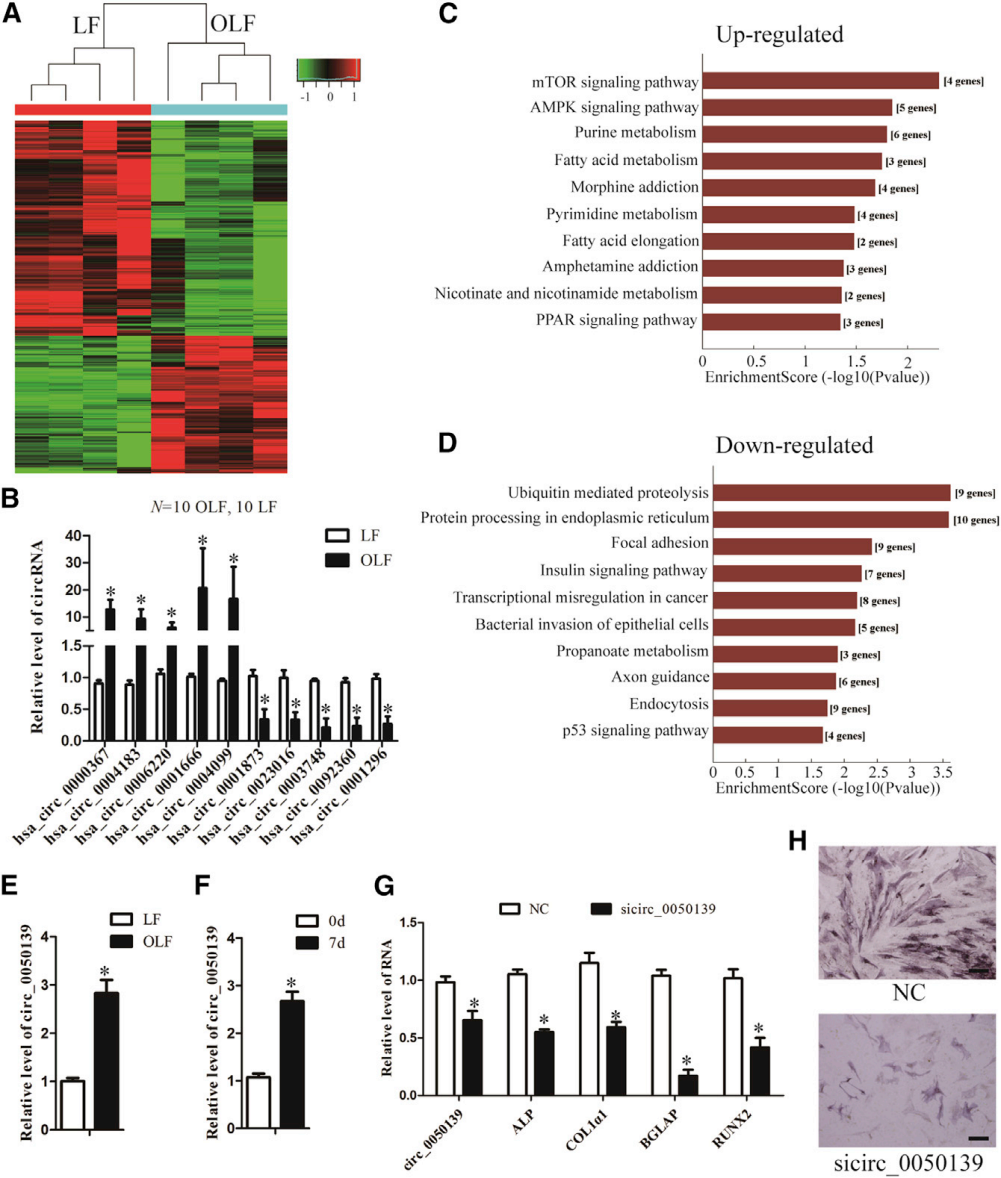
Differently Expressed circRNAs in OLF and the Function of circ_0050139 (A) Microarray analysis for circRNA was performed with RNA extracted from ossification (n = 4) and normal (n = 4) *ligamentum flavum*. Hierarchical cluster analysis of significantly differentially expressed circRNAs: bright green, under-expression; gray, no change; bright red, overexpression. (B) Differential expression of ten representative circRNAs was validated in human ossified and normal LF tissues by qPCR (n = 10 per group). (C and D) KEGG pathway enrichment analysis of linear counterparts of the upregulated (C) and downregulated (D) circRNAs with the top ten enrichment scores was performed. Both up- and downregulated circRNAs are significantly changed, with fold change > 2.0, p < 0.05 between OLF and normal LF tissues. (E and F) qPCR analysis of circ_0050139 was performed with total RNA isolated from ossified and normal LF (E) and hMSCs treated with osteogenic medium for 0 and 7 days (F). (G) hMSCs were transfected with sicirc_0050139 or the negative control and further cultured in osteogenic medium for 7 days. The RNA levels of circ_0050139, ALP, Col1α1, BGLAP, and RUNX2 were detected by qPCR. GAPDH was used as an internal control. (H) Representative images of ALP staining of hMSCs after transfection with sicirc_0050139 or the negative controls and further culture in osteogenic medium for 7 days. Scale bars, 400 μm. Data are presented as mean ± SEM; p values were analyzed by Student’s t test; *p < 0.05.

Arraystar human circRNA array analysis was also adopted for profiling human circRNA expression. The data showed that 244 circRNAs were upregulated and 383 circRNAs were downregulated in OLF tissues (fold change > 2, p < 0.05) (Figure 7A). The expression of ten randomly selected circRNAs was validated by qPCR, which was consistent with the results of the microarray (Figure 7B; Table S5).

It has been reported that circRNAs can function through their parental genes. Therefore, a correlation between the differentially expressed circRNAs and their linear counterparts was constructed, followed by GO and KEGG pathway analyses. However, the results of the GO and KEGG analyses showed that the top ten GO items and pathways are not obviously associated with ossification, apart from the mTOR signaling pathway (Figures 7C, 7D, S5A, and S5B). These data indicate that circRNA deregulation might be a side effect of the massive deregulation observed in OLF. To investigate whether the deregulated circRNAs, which might be a side effect of massive deregulation, are involved in OLF, we selected an upregulated circRNA, circ_0050139, which is a target of miR-19b-3p (Table S5). This molecule was confirmed to be overexpressed in OLF and hMSCs under osteogenic induction (Figures 7E and 7F). We further silenced it by siRNA to detect whether the expression of osteogenic differentiation-associated genes was affected. The results showed that knockdown of circRNA_0050139 reduced the transcripts of ALP, COL1α1, BGLAP, and RUNX2 and ALP staining in hMSCs under osteogenic induction (Figures 7G and 7H), confirming that this deregulated circRNA is involved in ossification.

### Construction of the miRNA-lncRNA-circRNA-mRNA network

**Figure 8:**
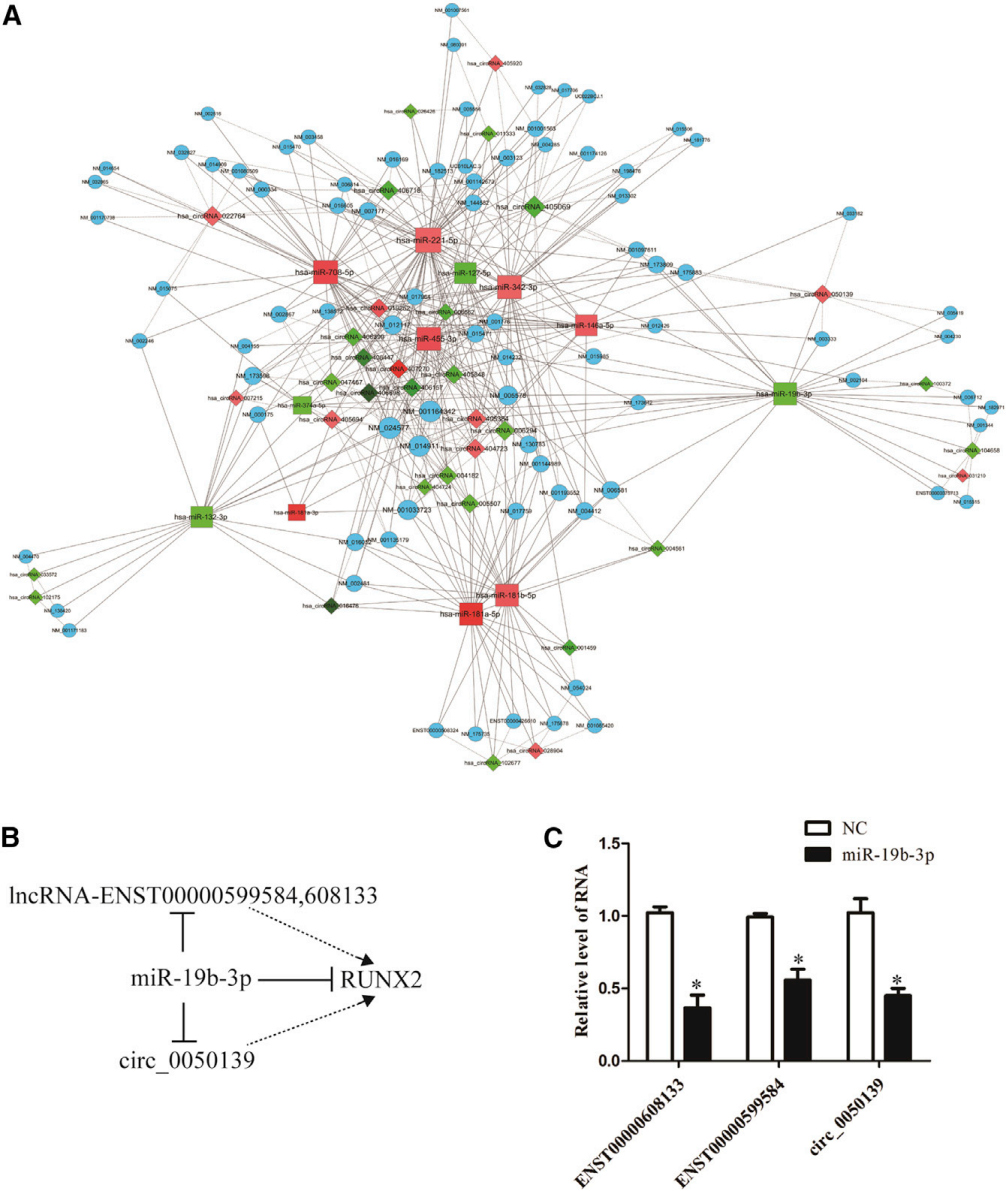
Integrated circRNA-microRNA-mRNA ceRNA Network Analysis (A) The competing endogenous RNA network has been based on circRNA-miRNA and miRNA-mRNA interactions. In this network, the circRNAs connect expression-correlated mRNAs via miRNAs. Square nodes represent miRNAs, diamonds represent circRNAs, and blue circles represent mRNAs. Red and green colors represent up- and downregulation, respectively. The shade of darkness represents fold change. The size of circles represents p values, with larger size owing smaller p value. Solid lines represent directed relationships, and dashed lines represent undirected relationships. (B) A simplified scheme was constructed, including the upregulated ENST00000608133, ENST00000599584, circ_0050139 and RUNX2 and the downregulated miR-19b-3p. (C) hMSCs were transfected with miR-19b-3p mimic or the negative control, and the RNA levels of ENST00000608133, ENST00000599584, and circ_0050139 were detected by qPCR. GAPDH was used as an internal control. Data are presented as mean ± SEM for at least triplicate experiments. The p values were analyzed by Student’s t test; *p < 0.05.

Furthermore, we predicted miRNAs that target the differentially expressed circRNAs and overlapped them with the deregulated miRNAs from the sequencing data to subsequently construct a circRNA-miRNA-mRNA relationship network, including the overlapped miRNAs, matching circRNA- and miRNA-targeted mRNAs (Figure 8A). The results showed that the miRNAs in this network, such as miR-181a, which has been reported to promote osteoblastic differentiation through repression of TGF-β signaling,^28^ and the mRNAs in this network, including LPP, ATF7, and ACTA1, are involved in the process of OLF.^29^, ^30^, ^31^

Given the evidence that the deregulated lncRNAs ENST00000608133 and ENST00000599584, which were actually targets of miR-19b-3p, are related to ossification by regulation, at least, of RUNX2, which is also regulated by miR-19b-3p and circ_0050139, we conclude that the deregulated miRNAs, lncRNAs, and circRNAs regulate ossification-associated genes (Figure 8B). Furthermore, we confirmed the co-regulation with experiments showing that overexpression of miR-19b-3p downregulates the levels of ENST00000608133, ENST00000599584, and circ_0050139 (Figure 8C).

## Discussion

Various cytokines, growth factors, and miRNAs have been shown to regulate the genes that orchestrate proliferation and differentiation in the process of ossification;^32^, ^33^ however, little information exists regarding the lncRNAs and circRNAs that regulate osteogenesis differentiation. In this study, we found that some mRNAs, lncRNAs, miRNAs, and circRNAs are altered during the OLF ossification process.

Serine peptidases (proteases) (for instance, human HTRA1 [high-temperature requirement serine protease A1]) positively regulate osteogenesis and mineralization of differentiating bone-forming cells through the modulation of ECM protein and have been implicated in musculoskeletal development.^34^ Figure 1C shows that the highest enrichment of upregulated genes concerning molecular function is associated with serine peptidase activity, which indicates that, in the process of ossification, serine peptidases promote bone formation. This result was linked with the rest of the data and the ossification process. Blood supply is essential for the function of ligaments, and angiogenesis is regulated by genes. Our chromatin immunoprecipitation (ChIP) data showed that the angiogenesis genes are downregulated (Figure S1A) (for instance, VEGF), indicating that reduced angiogenesis and the subsequent decreased blood supply in ligaments, but not in bone tissue, might cause ossification. On the other hand, the enrichment of blood vessels in normal ligaments might be more than that in ossified ligaments, with which the ChIP analysis was performed, and it showed that the angiogenesis genes are downregulated. We will conduct research to investigate this in a future study. In addition, very few genes have been identified and confirmed to be directly related to ligament function. This is also the reason why we have found an upregulation of bone-related genes but no downregulation of ligament-related genes.

The ossification of ligament tissue is a complex process characterized by gradually increasing osteophytes and regulated by signaling-regulatory networks, including growth factors, transcription factors, and ncRNAs, a number of which form feedback loops controlling the process of osteogenic differentiation.^35^ For instance, activated Wnt signaling through treatment with Wnt3a induces TAZ expression and increases its nuclear localization to stimulate osteogenic differentiation.^36^ Activated mTOR signaling promotes expression of the transcription factor RUNX2 to induce osteogenesis.^37^ TNF-α-activated NF-κB signaling increases the level of miR-150-3p, which directly targets the 3′ UTR of β-catenin mRNA and, in turn, represses its expression to inhibit the osteogenic differentiation.^38^ It has also been reported that there exists a feedback-regulatory loop consisting of miR-26a, GSK3β, and CCAAT/enhancer binding protein alpha (C/EBPα) that regulates osteogenesis.^39^

To date, the transcription profiling of OLF was mainly associated with genes encoding proteins, and much less was known about ncRNAs. Recent reports have demonstrated that ncRNAs participate in modulating numerous biological functions and pathological processes through regulating gene expression at the transcriptional and posttranscriptional levels.^40^, ^41^ For instance, a recent study has reported that the liver-enriched lnc-LFAR1 promotes hepatic stellate cell activation and TGFβ-induced hepatocyte apoptosis *in vitro* and aggravates both CCl_4_- and bile duct ligation-induced liver fibrosis by activating the TGF-β and Notch pathways.^12^ Also, it has been demonstrated that ciRS-7 can repress Alzheimer’s disease (AD) development by suppressing NF-κB protein synthesis and inducing its cytoplasmic localization, promoting UCHL1 expression and UCHL1-induced amyloid precursor protein (APP) and BACE1 ubiquitination and degradation.^13^ Moreover, primary ligament cells were preferentially used for transcription profiling in most OLF research.^42^, ^43^ In this study, for the first time, we used normal and ossified ligament tissues for transcription profiling, which reflect more closely the change in expression of ncRNAs and mRNA of OLF *in vivo*.

Despite their poor conservation and low levels of expression compared with protein-coding genes, lncRNAs are often regulated by transcription factors and are expressed in a cell- or tissue-specific manner.^12^, ^44^ In this study, 1,817 lncRNAs were upregulated and 750 lncRNAs were downregulated, suggesting that lncRNAs are more likely to be induced during the process of OLF. Further lncRNA subgroup analysis showed that the majority of differentially expressed lncRNAs were intergenic, which is consistent with the distribution of lncRNAs. In addition, from the lncRNA-mRNA co-expression network, we found that the mRNAs of MMP9 and BGLAP co-expressed with multiple lncRNAs, forming a complex network, which showed that a downregulated lncRNA, MIAT, was negatively correlated with the mRNAs of MMP9 and BGLAP, consistent with a previous study showing that the lncRNA MIAT inhibits osteogenic differentiation.^27^ In our study, most of the lncRNAs in the co-expression network were not yet annotated. It is very much worth it to perform further studies to reveal the underlying mechanisms of these lncRNAs.

circRNAs are drawing increased attention as one of the top hot ncRNAs. Evidence is emerging that circRNAs can participate in the regulation of gene expression in various ways. It has been reported that circRNAs can function through their parental genes. For instance, circular RNA that spans several exons of Ubiquitin (Ub) protein ligase 3 (E3) (cir-ITCH) can regulate the Wnt pathway by increasing the level of ITCH in esophageal squamous cell carcinoma.^45^ Additionally, many more circRNAs have been reported to harbor multiple miRNA binding sites, which seem to be a typical feature of this class of RNA molecules; this suggests that circRNA can act as a sponge of miRNA to regulate target genes. To date, various studies have made substantial progress regarding the function of CDR1 natural antisense transcript (NAT) to act as an miR-7 sponge *in vitro* and *in vivo* and, thus, have termed this circular transcript ciRS-7.^46^, ^47^ Therefore, in this study, a correlation between the differentially expressed circRNAs and their linear counterparts was constructed, followed by GO and KEGG pathway analyses. The results of the GO and KEGG analyses showed that the circRNA-correlated linear counterparts are not obviously associated with ossification, apart from the mTOR and peroxisome proliferators-activated receptor (PPAR) signaling pathways, indicating that the deregulated circRNAs might also be a side effect of the massive gene expression deregulation observed in OLF. However, in this study, as shown by the evidence of the selected circ_0050139, which regulates osteogenic differentiation-associated genes, it suggests that the differentially expressed circRNAs, a side effect of massive deregulation, may function by sponging the matched miRNA, thus releasing the miRNA inhibition instead of binding to their parental genes during the process of OLF.

In conclusion, our findings provide, for the first time, a systematic perspective on the potential function of ncRNAs and mRNAs during the process of OLF, illuminating the potential mechanism of OLF pathogenesis and providing new targets for OLF.

## Materials and Methods

### Sample Collection

Ossified and normal OLF tissues were obtained from OLF or spinal trauma patients who underwent posterior decompression laminectomy. We confirmed the diagnosis of OLF and spinal trauma by X-ray, computed tomography (CT), and MRI before the operation. The specimens were collected during surgery, rinsed with PBS, frozen in liquid nitrogen, and stored at −80°C for further study. Clinical samples were obtained after receiving informed consent from the patients. The study methodologies conformed to the standards set by the Declaration of Helsinki. This study was approved by the ethical review committee of Tianjin Medical University General Hospital.

### RNA Extraction and Quality Control

To isolate total RNA from the tissue, the frozen tissue was ground in liquid nitrogen, and total RNA was extracted with Trizol reagent (Life Technologies). Total RNA was extracted from cells with Trizol reagent. RNA quantity and quality were measured by NanoDrop ND-1000. RNA integrity was assessed by standard denaturing agarose gel electrophoresis and an Agilent 2100 Bioanalyzer. All RNA samples were stored at −80°C until further processing.

### Microarray Analysis

Arraystar Human lncRNA Microarray V4.0 was adopted for detection of lncRNAs and mRNA expression. Arraystar Human circRNA Array V2 (8x15K, Arraystar) was adopted for profiling circRNAs expression. All microarray analyses were performed by KangChen Bio-tech (Shanghai, China). Briefly, sample labeling and array hybridization were performed according to the Agilent One-Color Microarray-Based Gene Expression Analysis protocol (Agilent Technology) with minor modifications. The mRNA was purified from total RNA after removal of rRNA (mRNA-ONLY Eukaryotic mRNA Isolation Kit, Epicenter). Each sample was then amplified and transcribed into fluorescent cRNA along the entire length of the transcripts without 3′ bias utilizing a random priming method (Arraystar Flash RNA Labeling Kit, Arraystar). The labeled cRNAs were purified by RNeasy Mini Kit (QIAGEN). The concentration and specific activity of the labeled cRNAs (picomoles Cy3 per microgram cRNA) were measured by NanoDrop ND-1000. 1 μg of each labeled cRNA was fragmented by adding 5 μL 10× blocking agent and 1 μL of 25× fragmentation buffer, and then the mixture was heated at 60°C for 30 min. Finally, 25 μL 2× GE Healthcare hybridization buffer was added to dilute the labeled cRNA, and 50 μL of hybridization solution was dispensed into the gasket slide and assembled on the lncRNA expression microarray slide. The slides were incubated for 17 hr at 65°C in an Agilent hybridization oven. The hybridized arrays were washed, fixed, and scanned using the Agilent DNA microarray scanner (part number G2505C). Agilent Feature Extraction software (version 11.0.1.1) was used to analyze the acquired array images. Quantile normalization and subsequent data processing of lncRNA and mRNA were performed using the GeneSpring GX v12.1 software package (Agilent Technologies). A series of data processing, including quantile normalization of circRNA, was performed using the R software limma package. Differentially expressed lncRNAs, mRNAs, and circRNAs between the two groups were identified through fold change and p value and false discovery rate (FDR) filtering. Hierarchical clustering was performed to generate an overview of the characteristics of expression profiles based on values of significant differentially expressed transcripts.

### miRNA Sequence

Illumina NextSeq 500 was used for library construction following the manufacturer’s protocol. It included the following steps: 3′ adaptor ligation, 5′ adaptor ligation, cDNA synthesis, PCR amplification, and size selection of ∼135- to 155-bp PCR-amplified fragments (corresponding to ∼15- to 35-nt small RNAs). The libraries were denatured as single-stranded DNA molecules, captured on Illumina flow cells, amplified *in situ* as clusters, and finally sequenced for 51 cycles on Illumina NextSeq according to the manufacturer’s instructions. All profiling work was done with the help of KangChen Bio-tech (Shanghai, China). After sequencing, the Solexa CHASTITY quality filtered reads were harvested as clean reads. The adaptor sequences were trimmed, and the adaptor-trimmed reads (≥15 nt) were left. miRDeep2 software was used to predict the novel miRNAs with these trimmed reads. Then the trimmed reads were aligned to merged pre-miRNA databases (known pre-miRNA from miRBase v21 plus the newly predicted pre-miRNAs) using Novoalign software (v2.07.11) with, at most, one mismatch. We used the most abundant isomiR, the mature miRNA annotated in miRBase and all isoforms of miRNA (5p or 3p) to calculate miRNA expression. When comparing the differentially expressed miRNA profiles between two groups, the fold change and p value and FDR were calculated and used to identify significant differentially expressed miRNAs. Hierarchical clustering was performed to generate an overview of the characteristics of expression profiles based on values of significant differentially expressed transcripts.

### Correlation and Co-expression Analysis

The co-expression analysis was based on calculating the Pearson correlation coefficient (PCC) between coding genes and noncoding transcripts according to their expression levels. The absolute values of parameter PCC ≥ 0.968 and p < 0.05 were recommended and retained for further analysis.

### ceRNA Network Analysis

circRNAs-miRNAs and miRNAs-mRNAs whose expression levels shared a meaningful correlation were subjected to the analysis. The potential miRNA response elements were searched on the sequences of circRNAs and mRNAs, and the overlap of the same miRNA seed sequence binding site both on the circRNAs and the mRNA predicted circRNA-miRNA-mRNA interaction.

### GO and KEGG Pathway Analysis

GO analysis was conducted to construct meaningful annotation of genes and gene products in a wide variety of organisms. The ontology has covered domains of biological processes (BPs), cellular components (CCs), and molecular functions (MFs). The-log10 (p value) denotes the enrichment score, representing the significance of GO term enrichment among differentially expressed genes. KEGG pathway analysis was also performed to harvest pathway clusters covering our knowledge of the molecular interaction and reaction networks in differentially regulated gene profiling. The −log10 (p value) denotes the enrichment score, showing the significance of the pathway correlations. The p value was corrected by FDR.

### Real-Time qPCR

Total RNA was extracted from OLF tissues with Trizol reagent (Life Technologies, Grand Island, NY, USA). For qPCR of mRNA, lncRNA, and circRNA, total RNA was digested with DNase I (Takara, Dalian, China). Briefly, the 10-μL reverse transcription (RT) reaction (1 μg RNA, 1 μL buffer, 1 μL DNase1, and water) was incubated for 15 min at 37°C, followed by addition of 1 μL of EDTA, incubation for 10 min at 65°C, and then maintenance at 4°C. Next, the first-strand cDNA was synthesized using avian myeloblastosis virus (AMV) reverse transcriptase (Thermo Fisher Scientific, Basingstoke, UK) according to the manufacturer’s instructions. For real-time PCR, all reactions were performed in triplicate with SYBR Green Master Mix (Takara, Dalian, China) under the following conditions: 15 min at 95°C for initial denaturation, followed by 40 cycles of segments of 95°C for 30 s and 60°C for 30 s in the Light Cycler 96 real-time PCR system (Roche, Mannheim, Germany). The expression levels of the housekeeping gene GAPDH were used to normalize the expression levels of the genes of interest. For qPCR of miRNA, reverse transcribed using The First-strand cDNA Synthesize (Sangon, B532451). For real-time PCR, all reactions were performed in triplicate with SYBR Green Master Mix (Takara, Dalian, China) under the following conditions: 15 min at 95°C for initial denaturation, followed by 40 cycles of segments of 95°C for 30 s and 60°C for 30 s in the Light Cycler 96 real-time PCR system (Roche, Mannheim, Germany). The expression levels of U6 were used to normalize the expression levels of the genes of interest. The primers used in the real-time PCR are listed in Table S6.

### Cell Cultures and Treatment

The primary hMSCs (Cyagen Biosciences) were cultured in alpha modified Eagle’s medium (Invitrogen) supplemented with 1% penicillin and streptomycin (Gibco) and 10% fetal bovine serum (FBS, Gibco). Cells were cultured under conditions of 5% CO_2_. Confluent cells were digested with 0.25% trypsin including 10 mM EDTA, re-suspended in corresponding medium, and plated into plates with a suitable density. To induce osteoblast differentiation, 50 μg/mL of ascorbic acid (Sigma), 5 mM β-glycerophosphate (Sigma), and 10 nM dexamethasone (Sigma) were add to the culture medium.

### Transfection Assay

hMSCs were cultured overnight until 50% confluence and then transfected with siRNA (supplied by GenePharma) using Lipofectamine 2000 (Invitrogen) according to the instructions. After 4 hr of transfection, the medium containing transfection reagent was replaced by fresh induction medium. After culturing for an additional 48 hr, cells were used for further experiments. The siRNA sequences for lncRNAs and circRNA are listed in Table S6.

### Alkaline Phosphatase Staining

Alkaline phosphatase staining was monitored with the 5-bromo-4-chloro-3-indolyl phosphate (BCIP) and NBT Alkaline Phosphatase Color Development Kit (C3206, Beyotime Institute of Biotechnology) in accordance with the instructions. Briefly, cells were fixed by immersion in 4% paraformaldehyde at 4°C for 30 min and rinsed in PBS for 5 min. The alkaline phosphatase stain was then added to the plates for 30 min while protecting the plates from light. The pictures were monitored by microscopy after rinsing in deionized water for 5 min.

### Statistical Analysis

Data are expressed as mean ± SEM. All the statistical analyses were performed with SPSS 13.0 (IBM, Armonk, NY, USA). Statistical analysis was performed using either Student’s t test (two-group comparison) or one-way analysis of variance (more than two groups), followed by *post hoc* comparison, and differences with p < 0.05 were considered significant.

### Accession Numbers

The accession numbers for the lncRNA and mRNA microarray, circRNA microarray, and miRNA-seq data reported in this paper are NCBI GEO: GSE106253, GSE106255, and GSE106256.

## Author Contributions

W.H. and Y.X. conceived and designed the study. Y. Han, Y. Hong, and L.L. performed the majority of the experiments. Z.S., X.H., and T.C. gave technical support and conceptual advice. W.H., Y. Han, and K.Z. analyzed the data and wrote the manuscript. L.L., T.L., H.X., and Y.T. collected clinical samples. Y. Han, Y. Hong, K.Z., Z.Z., J.W., Q.L., M.Z., Y.X., and W.H. contributed reagents, materials, and analysis tools.

## Conflicts of Interest

We declare no competing interests.

## Supplemental Information

Document S1. Figures S1–S5 and Tables S1–S6

Document S2. Article plus Supplemental Information
