# Supplementary material for: A Transcriptome-Level Study Identifies Changing Expression Profiles for Ossification of the Ligamentum Flavum of the Spine
Source: Mol Ther Nucleic Acids. 2018 Aug 7;12:872–83. doi: 10.1016/j.omtn.2018.07.018 (PMC6120750; doi:10.1016/j.omtn.2018.07.018)
Supplement: Document S1. Figures S1–S5 and Tables S1–S6 [file mmc1.pdf]

OMTN, Volume *12*

Supplemental Information

A Transcriptome-Level Study Identifies Changing Expression Profiles for Ossification of the *Ligamentum Flavum* of the Spine

Yawei Han, Yuheng Hong, Liandong Li, Tengshuai Li, Zhen Zhang, Jingzhao Wang, Han Xia, Yutao Tang, Zhemin Shi, Xiaohui Han, Ting Chen, Qi Liu, Mengxia Zhang, Kun Zhang, Wei Hong, and Yuan Xue

Supplementary Figures

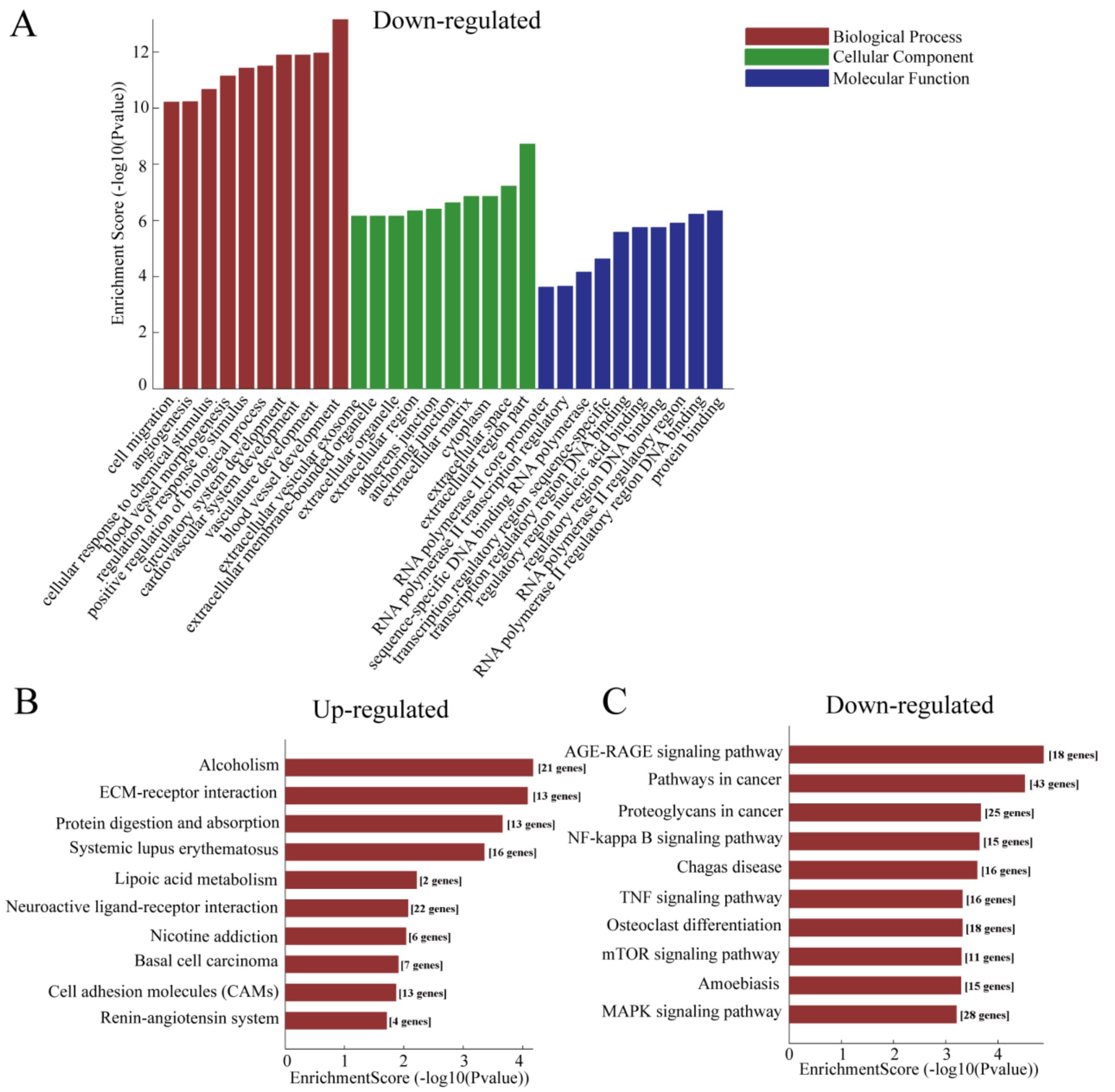

Supplementary Figure 1. GO and KEGG pathway analysis of differentially expressed mRNAs. (A) GO annotation of the down-regulated mRNAs with top ten enrichment score covering domains of biological processes, cellular components and molecular functions were performed. (B-C) KEGG pathway enrichment analysis of up- (B) and down-regulated (C) mRNAs with top ten enrichment score was performed. Both up- and down-regulated mRNAs are significantly changed with fold change > 2.0, $p < 0.05$ between OLF and normal LF tissues.

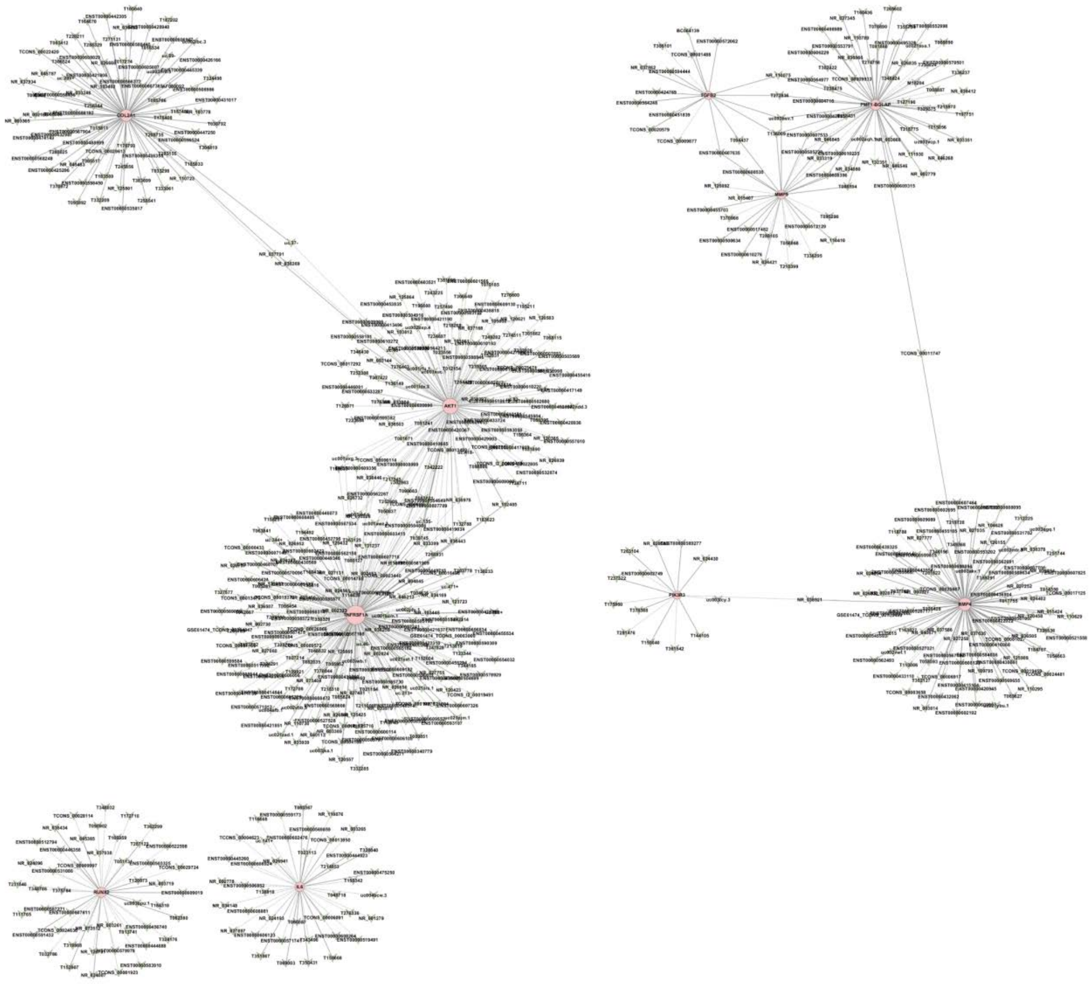

Supplementary Figure 2. Co-expression network of ten significantly deregulated mRNAs and their co-expressed lncRNAs. The coding genes, including RUNX2, MMP9, BGLAP, IL6, COL2α1, AKT1, TNFRSF1A, TGFB2, BMP4 and PIK3R3, and their co-expressed lncRNAs, that have Pearson correlation coefficients≥0.968 were chosen to draw the network using Cytoscape. V nodes represent lncRNA and circle nodes represent mRNAs. Solid lines represent positive relationship and dash lines represent negative relationship.

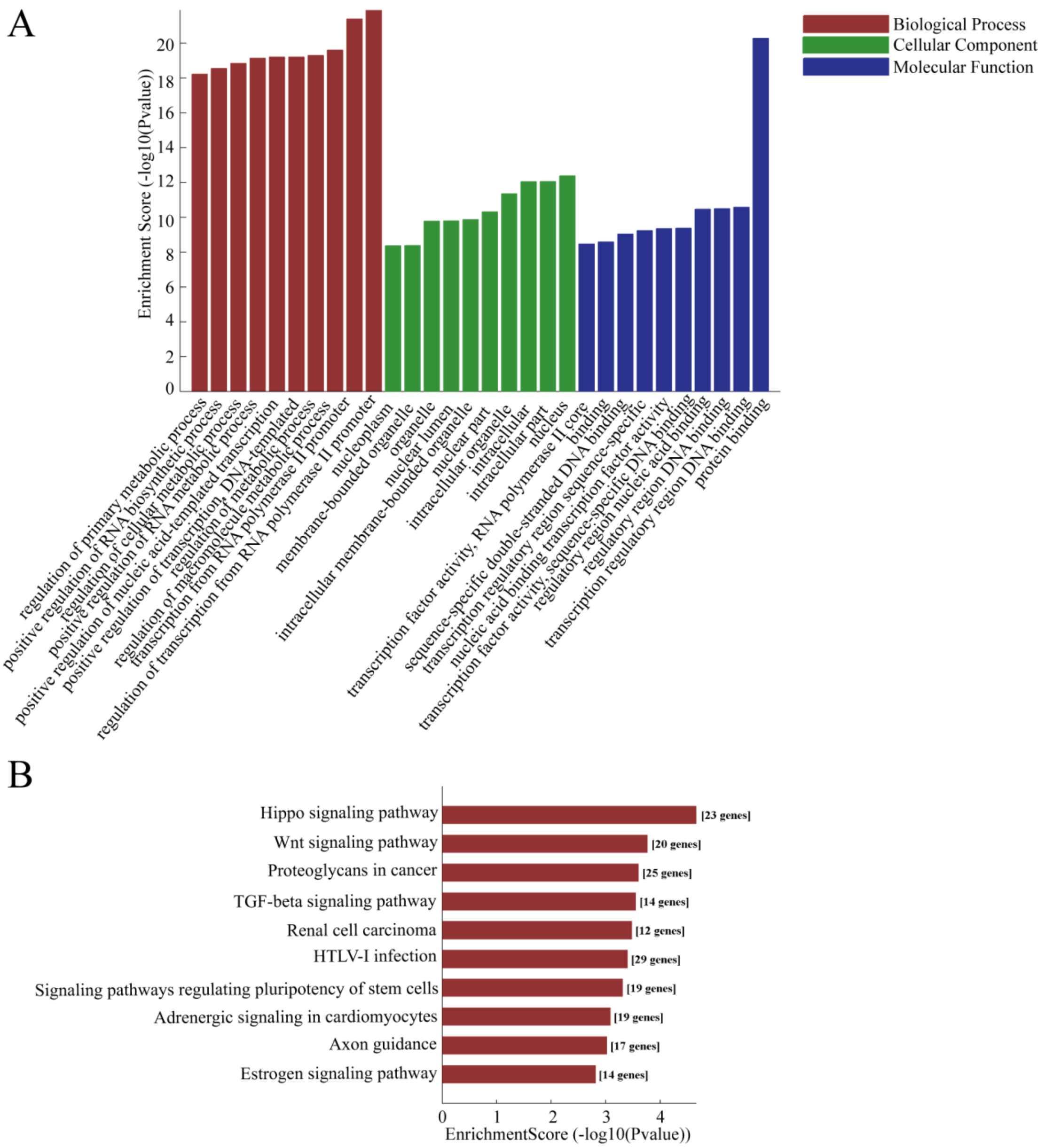

Supplementary Figure 3. GO and KEGG pathway analysis of miRNAs target genes. (A) GO annotation of all targeted mRNAs of deregulated miRNAs with top ten enrichment score covering domains of biological processes, cellular components and molecular functions was performed. (B) KEGG pathway enrichment analysis of targeted mRNAs of all deregulated miRNA with top ten enrichment score was performed. Both up- and down-regulated miRNAs are significantly changed with fold change>2.0, p<0.05 between OLF and normal LF tissues.

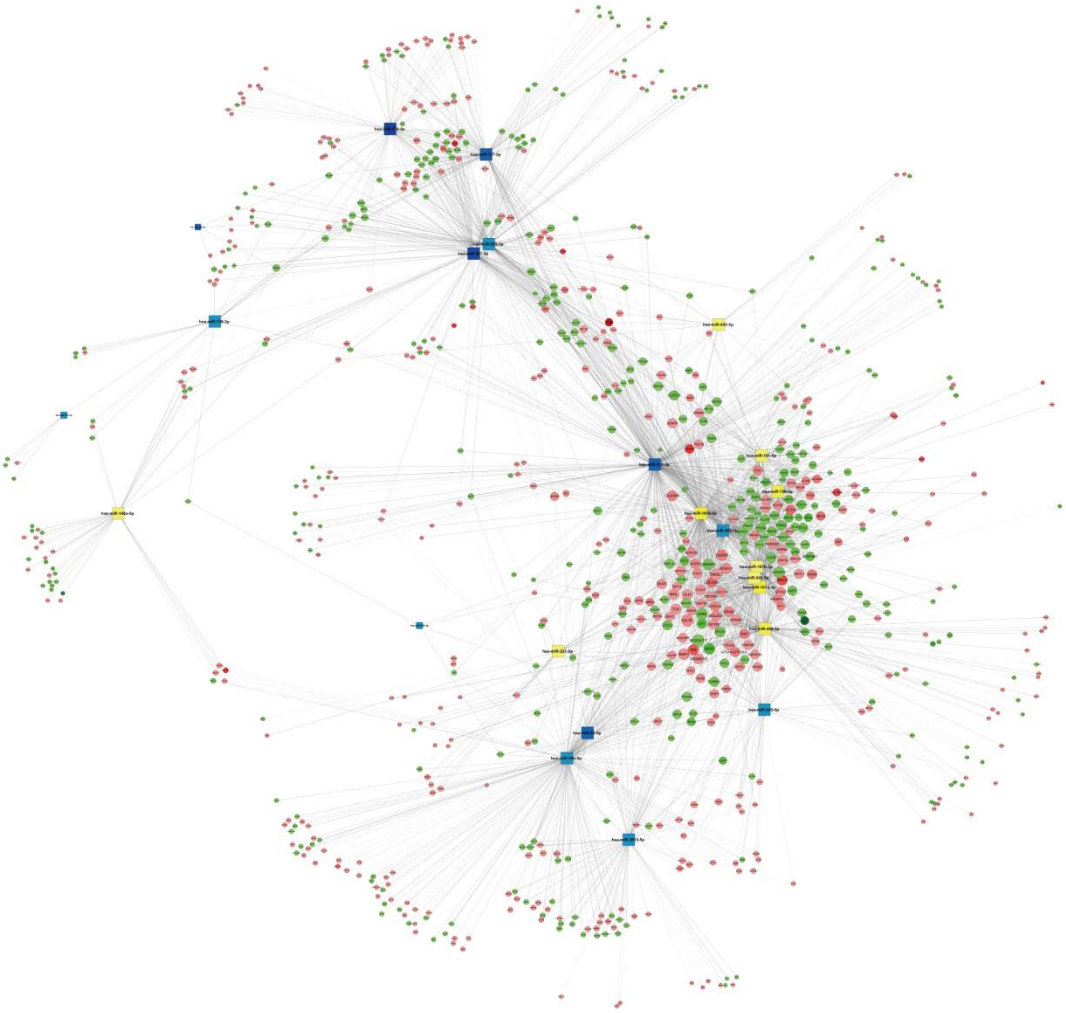

Supplementary Figure 4. The miRNA-mRNA interacting network. The differentially expressed mRNAs identified previously (Figure 1A) overlap with the predicted miRNAs targets, and only the genes that negatively correlated with its corresponding miRNAs are included. These inversely correlated miRNA/mRNA pairs were sorted to construct the networks. Circle nodes represent mRNAs and square nodes represent miRNAs. Red and yellow color represent up regulation, and green and blue color represent down regulation respectively. The shade darkness represents fold change. The size of circle represents *p* value with larger size owing smaller *p* value. Solid lines represent positive relationship and dash lines represent negative relationship.

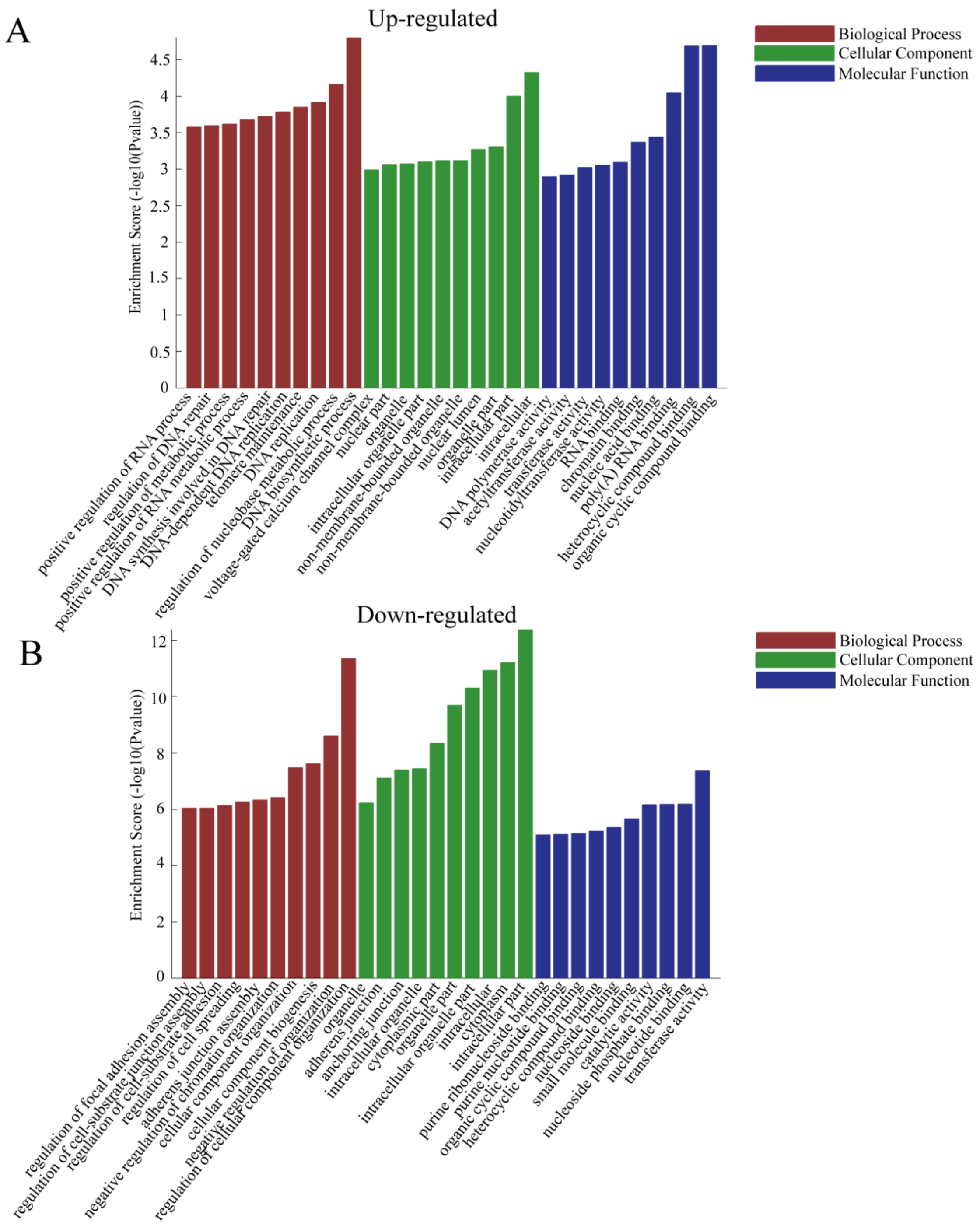

Supplementary Figure 5. GO and KEGG pathway analysis of circRNAs-related linear counterparts. (A-B) GO annotation of the linear counterparts of up- (A) and down-regulated (B) circRNAs with top ten enrichment score covering domains of biological processes, cellular components and molecular functions was performed. Both up- and down-regulated miRNAs are significantly changed with fold change > 2.0, $p < 0.05$ between OLF and normal LF tissues.

Supplementary Table 1. The information about the patients

Serial number	Age	Sex	Disease	Tissue
OLF-1#	56	F	Spinal stenosis	Ossified ligamentum flavum
OLF-2#	57	M	Spinal stenosis	Ossified ligamentum flavum
OLF-3#	60	M	Spinal stenosis	Ossified ligamentum flavum
OLF-4#	68	M	Spinal stenosis	Ossified ligamentum flavum
LF-1#	42	M	Spinal trauma	Normal ligamentum flavum
LF-2#	57	M	Spinal trauma	Normal ligamentum flavum
LF-3#	73	M	Spinal trauma	Normal ligamentum flavum
LF-4#	40	F	Spinal trauma	Normal ligamentum flavum

Supplementary Table 2. The deregulated expression of mRNAs in microarray in OLF

Gene Symbol	Seqname	Regulation	Fold Change	*p* value
AK5	NM_174858	up	24.6237614	0.002139029
COL2A1	NM_001844	up	24.3281001	0.000183825
PMF1-BGLAP	NM_001199661	up	14.0520984	0.001964946
RUNX2	NM_001024630	up	10.5248663	0.04779347
BMP4	NM_001202	up	4.7992443	0.00207799
IL6	NM_000600	down	62.2617382	0.027100353
CRACR2B	NM_001286606	down	20.0370609	0.018754584
SAA1	NM_000331	down	14.8008962	0.00492719
G0S2	NM_015714	down	13.6180691	0.007933331
ATF3	NM_001674	down	7.0536263	0.002535643

Supplementary Table 3. The deregulated expression of lncRNAs in microarray in OLF

Gene Symbol	Seqname	Regulation	Fold Change	*p* value
RP11-644F5.16	ENST00000556606	up	48.3224434	0.002456396
LOC101927269	NR_110075	up	31.1198732	0.001515081
LINC00347	NR_034024	up	17.0523229	0.001624259
CTD-2249K22.1	ENST00000512859	up	2.275214	0.013428992
LINC01135	NR_034015	up	2.9781847	0.000480586
LOC100505817	NR_038340	down	25.5974901	0.027545948
RP11-109E24.1	ENST00000511279	down	11.7276114	0.004813486
AQP7P3	NR_026558	down	9.1849429	0.010875482
RP11-720L8.1	ENST00000561054	down	6.9156078	0.001576764
lncMIAT	NR_033319	down	2.6070678	0.015431081
RP11-523G9.3	ENST00000608133	up	2.5794996	0.029309278
CTB-180A7.6	ENST00000599584	up	2.5120166	0.013285599

Supplementary Table 4. The deregulated expression of miRNAs in sequencing in OLF

Gene Symbol	Regulation	Fold Change	*p* value
hsa-miR-181a-5p	up	5.013659	9.87557E-05
hsa-miR-181a-3p	up	4.609562	0.004157204
hsa-miR-708-5p	up	3.632302	0.009366564
hsa-miR-181b-5p	up	2.738144	0.006102139
hsa-miR-146a-5p	up	2.538066	0.023043047
hsa-miR-889-3p	down	2.949365297	0.007410704
hsa-miR-32-5p	down	3.371430498	0.03504732
hsa-miR-379-5p	down	4.340579207	0.02562263
hsa-miR-novel-chrX_47539	down	5.016881807	0.007573798
hsa-miR-381-3p	down	5.930389093	6.51417E-05
hsa-miR-19b-3p	down	2.069497878	0.006346186

Supplementary Table 5. The deregulated expression of circRNAs in microarray

Gene Symbol	Regulation	Fold Change	*p* value
hsa_circ_0000367	up	30.7576643	2.25293E-06
hsa_circ_0004183	up	20.0548723	0.002363038
hsa_circ_0006220	up	16.0456877	3.82059E-06
hsa_circ_0001666	up	37.4327367	4.4194E-06
hsa_circ_0004099	up	28.1184624	0.002744821
hsa_circ_0001873	down	4.6512421	0.000257104
hsa_circ_0023016	down	15.5507265	0.028144848
hsa_circ_0003748	down	15.0729102	0.021007797
hsa_circ_0092360	down	14.1954431	0.036578546
hsa_circ_0001296	down	13.4871247	0.016418551
hsa_circ_0050139	up	2.3049384	0.003656394

Supplementary Table 6. Primers for qPCR, Related to Experimental Procedures

qPCR primers for analysis of mRNA

Gene symbol	Forward 5' - 3'	Reverse 5' - 3'
AK5	CCTGATGAAGAGGGCATTGT	TGATTAGCACAAGCCAGGAA
COL2A1	AAGGGTGATCGTGGTGAGAC	GCACCAGCTTCTCCTCTGTC
CRACR2B	ACAAGGAGGCTAAGGGCTTC	GGTGAGGAAGCCAGTGTGA
Runx2	TGCGGCCGCCCCACGACAA	ACCCGCCATGACAGTAACCACAGT
BGLAP	CACTCCTCGCCCTATTGGC	CCCTCCTGCTTGGACACAAAG
SAA1	CGGCTCAGACAAATACTTCCA	CACCATGGCCAAAGAATCTC
IL6	CTCAATATTAGAGTCTCAACCCCCA	GAGAAGGCAACTGGACCGAA
BMP4	CGTCCAAGCTATCTCGAGCC	CATAGGTCCCTGCAGTAGCG
G0S2	GCCGTGCCACTAAGGTCATT	GATCAGCTCCTGGACCGTTT
ATF3	TGATGCTTCAACACCCAGGC	GGATGGCAAACCTCAGCTCT
GAPDH	ACCCAGAAGACTGTGGATGG	TTCAGCTCAGGGATGACCTT

qPCR primers for analysis of lncRNA

Seqname	Forward 5' - 3'	Reverse 5 '- 3'
ENST00000556606	AGAGGCTGGGCTACAGAAGAA	GAGGCTCTGCTCTTGTGCG
ENST00000512859	GTGTGGGGTACGTTCACCTT	ACGGGCAGGTTTATTTGGGT
ENST00000511279	TGCCAGATTCATGATGCCAC	GCTCCTCAGTTGTAGCCTCTC
ENST00000561054	TGGGGAACCAGCAAACTCAT	ACTGACAAACTCCCACGCTG
NR_033319	AGGATTCCTTGCATGGGGTG	CCCAAGAAGCACCGTGAGAT
NR_110075	GAGCTGTAGCAGAAAAGCAAGT	TCCAATCCATTGTCCACGCT
NR_034024	GAGGAGGGGCCTTTCATAGC	CAATTACCACAGGCGGACCT
NR_034015	AGCGCTAGACACATGGAACC	TTCCAGCGCGACTGTTGTTA
NR_038340	GGTGACCTGGGCTCCTTATG	CTGCAAGTGTCTTCCTGTGGA
NR_026558	CATGGTTCAAGCATCTGGGC	TAGAGCCTGCATCGTGGTTC
ENST00000608133	AGAGGCATTTGTCGGTAGTTTT	TCTGATGGGCATCCACACATT
ENST00000599584	CCGAGGAGGGTTTCTCTAGC	GTCCCTCCACAGCCCTTAAC

qPCR primers for analysis of miRNA

Gene symbol	primers
hsa-miR-181a-5p	AACATTCAACGCTGTCGGTGA
hsa-miR-181a-3p	ACCATCGACCGTTGATTGTAC
hsa-miR-708-5p	AAGGAGCTTACAATCTAGCTGG
hsa-miR-181b-5p	AACATTCATTGCTGTCGGTGG
hsa-miR-146a-5p	ATGAGAACTGAATTCCATGGGTT
hsa-miR-379-5p	TGGTAGACTATGGAACGTAGG
hsa-miR-novel-chrX_47539	ACTCGATGTTGGATCAGGACA
hsa-miR-381-3p	TATACAAGGGCAAGCTCTCTGT
hsa-miR-889-3p	CGTTAATATCGGACAACCATTGT
hsa-miR-32-5p	CGTATTGCACATTACTAAGTTGCA
hsa-miR-19b-3p	TGTGCAAATCCATGCAAAACTGA

qPCR primers for analysis of circRNA

Gene symbol	Forward 5' - 3'	Reverse 5 '- 3'
hsa_circ_0000367	AGACTGGCGTGAAACCTTCC	CGTATGGAATGGACCTGGACA
hsa_circ_0001873	CTGAGCTCATGTGAGGGTGA	TCGTGCGGATGATCGGGATA
hsa_circ_0004183	CGTCCATTCCACGAGGTTCT	TTCAAGGCAGCCAGCATGTC
hsa_circ_0006220	TGCATCTACCCTGCTGAACCT	TTGGCTACATCCTGCAGTGAAA
hsa_circ_0023016	GAGAGTCTCACTGCTCCAGGG	CCACACACATCCCACTACCC
hsa_circ_0003748	TGTGGCATGCAGCCATAAGTT	GCTGGTGACATTTCATGCTC
hsa_circ_0001666	GCCTAGCTGTCAAGGAGTGG	CTTGCAAACCTCTCACACCCA
hsa_circ_0004099	AGGTTTTTGAACTGCTCGGGG	GGCACCATCCCTGGCTTTAGA
hsa_circ_0092360	GGCCGGAAGTGACCGATTTC	CAGCCGTGTTCCCAATTCGT
hsa_circ_0001296	GTGTGCTGTGATGAGGAAGATGA	AGCACGCCGTTCAATCACAT
hsa_circ_0050139	CCCCATGACTCAGGGTTTTGA	TCTTCTTGCGGCAGTTGACA

siRNA sequences for lncRNAs and circRNA

Gene symbol	Forward 5' - 3'	Reverse 5 '- 3'
siENST00000608133	GAAGGCACCAUUUAACAAATT	UUUGUUAAAUGGUGCCUUCTT
siENST00000599584	GGCUUUGGAUGAGACCGUATT	UACGGUCUCAUCCAAAGCCTT
sihsa_circ_0050139	GCUGCUCAGUGCUAUGCUCTT	GAGCAUAGCACUGAGCAGCTT
