# Supplementary material for: A Transcriptome-Level Study Identifies Changing Expression Profiles for Ossification of the Ligamentum Flavum of the Spine
Source: Mol Ther Nucleic Acids. 2018 Aug 7;12:872–83. doi: 10.1016/j.omtn.2018.07.018 (PMC6120750; doi:10.1016/j.omtn.2018.07.018)
Supplement: Document S2. Article plus Supplemental Information [file mmc2.pdf]

Molecular Therapy
Nucleic Acids
Original Article

AMERICAN SOCIETY of GENE & CELL THERAPY

A Transcriptome-Level Study Identifies Changing Expression Profiles for Ossification of the *Ligamentum Flavum* of the Spine

Yawei Han,[1,4] Yuheng Hong,[2,4] Liandong Li,[3,4] Tengshuai Li,[3] Zhen Zhang,[1] Jingzhao Wang,[1] Han Xia,[3] Yutao Tang,[3] Zhemin Shi,[1] Xiaohui Han,[1] Ting Chen,[1] Qi Liu,[1] Mengxia Zhang,[1] Kun Zhang,[1] Wei Hong,[1] and Yuan Xue[3]

[1]Department of Histology and Embryology, School of Basic Medical Sciences, Tianjin Medical University, Tianjin, China; [2]School of Medical Imaging, Tianjin Medical University, Tianjin, China; [3]Department of Orthopaedics, Tianjin Medical University General Hospital, Tianjin, China

Ossification of the *ligamentum flavum* (OLF) is a common spinal disorder that causes myelopathy and radiculopathy. Non-coding RNAs (ncRNAs) are involved in numerous pathological processes; however, very few ncRNAs have been identified to be correlated with OLF. Here we compared the expression of lncRNA, mRNA, circRNA, and microRNA in OLF tissues from OLF patients and healthy volunteers through mRNA, lncRNA, and circRNA microarrays and microRNA sequencing. A total of 2,054 mRNAs, 2,567 lncRNAs, 627 circRNAs, and 28 microRNAs (miRNAs) were altered during the process of OLF. qPCR confirmed the differential expression of selected mRNAs and ncRNAs. An lncRNA-mRNA co-expression network, miRNA-mRNA target prediction network, and competing endogenous RNA (ceRNA) network of circRNA-miRNA-mRNA were constructed based on a correlation analysis of the differentially expressed RNA transcripts. Subsequently, gene ontology (GO) and Kyoto Encyclopedia of Genes and Genomes (KEGG) pathway analyses for the differentially expressed mRNAs and the predicted miRNAs target genes were performed. In addition, a deregulated miRNA-19b-3p-based miRNA-circRNA-lncRNA-mRNA network was confirmed, by gain-of-function and loss-of-function experiments, to function in the process of ossification. Taken together, this study provides a systematic perspective on the potential function of ncRNAs in the pathogenesis of OLF.

INTRODUCTION

Ossification of the *ligamentum flavum* (OLF) of the spine is characterized by ectopic bone formation in the OLF and is highly prevalent in the population of East Asia.[1,2] The ossified ligaments form osteophytes that gradually increase in size, which, in turn, causes compression of the spinal cord and may lead to severe neurological symptoms. Many factors, such as genetic background, dietary habits, metabolic abnormalities, and mechanical stress, contribute to OLF.[3–5] Additionally, many protein-coding genes, especially RUNX2, have been reported to be associated with the development of OLF and other ossification-related events, such as chondrocyte maturation, endochondral ossification, and fetal bone development.[6,7] However, the exact pathogenesis of OLF still remains unclear.

The number of human protein-coding genes is less than 2% of the whole genome sequence, whereas the vast majority of transcripts consist of non-coding RNAs (ncRNAs), including microRNA (miRNA), circular RNA (circRNA), and long ncRNA (lncRNA).[8,9] With multiple and diverse targets, ncRNAs are involved in numerous biological functions and pathological processes, including development, proliferation, apoptosis, survival, differentiation, and carcinogenesis.[10–13] The specific contribution of selected ncRNAs to osteogenic differentiation has been described.[14] Recent studies reported that the process of osteogenic differentiation is governed by distinct miRNAs and lncRNAs. For instance, miR-214 has a crucial role in suppressing bone formation by inhibiting osteoblast activity through targeting ATF4.[11] Moreover, it has been reported that bone mesenchymal stem cell (BMSC)-specific inhibition of miR-188 by intra–bone marrow injection of antagomiR-188 increases bone formation and decreases bone marrow fat accumulation in aged mice.[15] Additional studies reported that H19 mediates BMP9-induced osteogenic differentiation of mesenchymal stem cells (MSCs) through Notch signaling[16] and that MEG3 inhibits adipogenesis and promotes osteogenesis of MSCs via miR-140-5p.[17] Although the field is developing, little is known about the functions of lncRNAs and circRNAs in OLF. Specifically, studies to date lack accurate lncRNA and circRNA profiling of OLF tissues.

In this study, we investigated the differentially expressed patterns of miRNA using miRNA sequencing and the differentially expressed patterns of lncRNA, mRNA, and circRNA using a microarray in OLF tissues. The randomly selected differential expression of representative miRNAs, lncRNAs, mRNAs, and circRNAs were further

Received 20 December 2017; accepted 31 July 2018;
https://doi.org/10.1016/j.omtn.2018.07.018.

[4]These authors contributed equally to this work.

Correspondence: Wei Hong, Department of Histology and Embryology, School of Basic Medical Sciences, Tianjin Medical University, No. 22 Qixiangtai Road, Heping District, Tianjin, China.
E-mail: hongwei@tmu.edu.cn
Correspondence: Yuan Xue, Department of Orthopaedics, Tianjin Medical University General Hospital, Tianjin, China.
E-mail: xueyuan@medmail.com.cn

 © 2018 The Authors.
This is an open access article under the CC BY license (http://creativecommons.org/licenses/by/4.0/).

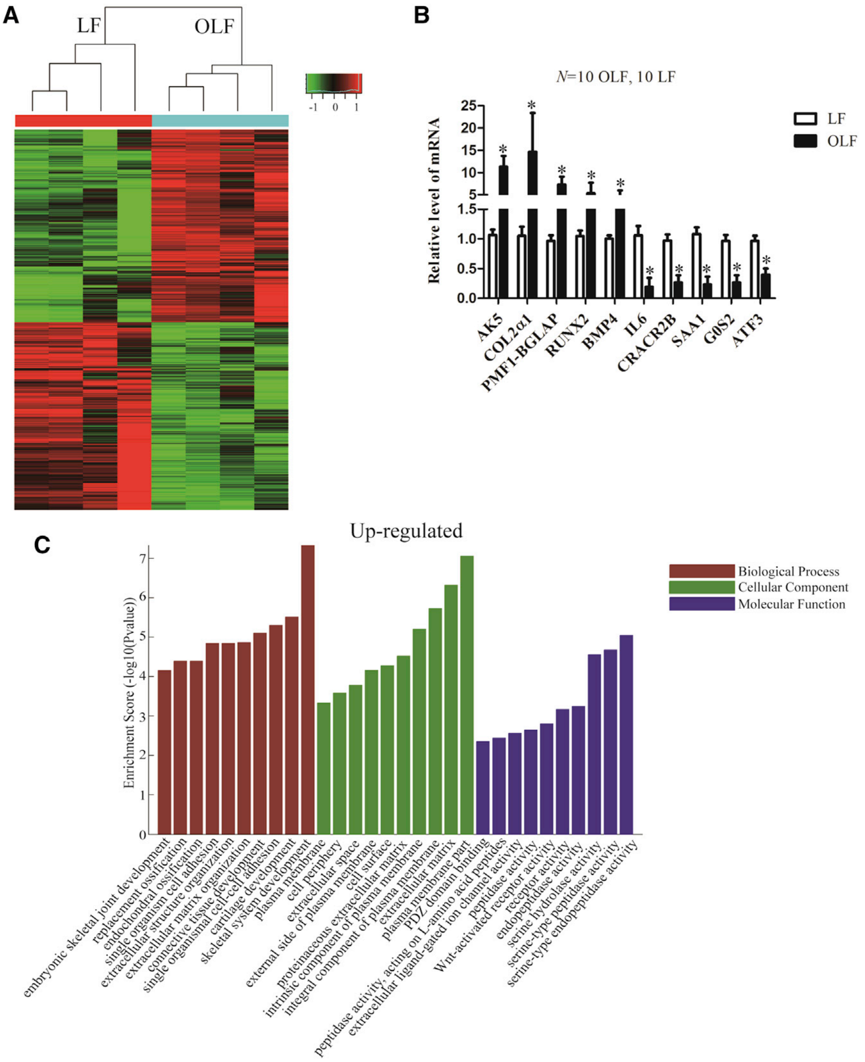

Figure 1. Expression Profile of mRNAs in OLF and Normal LF

(A) Microarray analysis for mRNA was performed with RNA extracted from ossified (n = 4) and normal (n = 4) *ligamentum flavum*. Hierarchical cluster analysis of significantly differentially expressed mRNAs: bright green, under-expression; gray, no change; bright red, over-expression. (B) Ten differentially expressed representative mRNAs were validated in OLF and normal LF tissues by qPCR (n = 10 per group). GAPDH was used as an internal control. (C) GO annotation of the linear counterparts of upregulated mRNAs with the top ten enrichment scores covering domains of biological processes, CCs, and MFs was performed. Data are presented as mean ± SEM; p values were analyzed by Student's t test; *p < 0.05.

confirmed using qPCR. Additionally, we constructed a co-expression network of lncRNA-mRNA, a target prediction network of miRNA-mRNA, and a competing endogenous RNA (ceRNA) mechanism network of circRNA-miRNA-mRNA based on a correlation analysis between the differentially expressed RNA transcripts for the first time in OLF. Subsequently, the differentially expressed mRNAs and the predicted miRNAs target genes followed by gene ontology (GO) and Kyoto Encyclopedia of Genes and Genomes (KEGG) pathway analyses were performed. This study not only provides a systematic perspective on the potential function of ncRNAs and mRNAs during the process of OLF but also illuminates the potential mechanism of OLF pathogenesis and provides new targets for OLF.

RESULTS

Identification of Differentially Expressed mRNAs in OLF

First, we collected ossified and normal LF tissues from patients during surgery; information about the patients and the section of ossification are shown in Table S1. The mRNA expression profiles were detected in 4 OLF and 4 normal LF tissues, and hierarchical clustering (Figure 1A) was performed to show distinguishable mRNA expression patterns among samples. From the mRNA expression profiling data, 2,054 mRNAs were differentially expressed in OLF tissues (fold change > 2, $p < 0.05$), of which 1,041 mRNAs were upregulated, and 1,013 mRNAs were downregulated. Five upregulated and five downregulated mRNAs were randomly selected to verify the microarray data in extension samples of OLF and normal LF tissues by qRT-PCR, and the results were consistent with the microarray data (Figure 1B; Table S2).

Subsequently, we conducted the GO and KEGG pathway analyses, and the results showed that the top ten most enriched GO terms of the upregulated genes include endochondral ossification, replacement ossification, extracellular matrix, and Wnt-activated receptor activity (Figure 1C), which are associated with ossification and osteogenic differentiation. The top ten most enriched GO terms of the downregulated genes are shown in Figure S1A. KEGG pathway analysis revealed that the pathways of upregulated genes (Figure S1B) and downregulated genes (Figure S1C) were associated with osteogenic differentiation, including extracellular matrix (ECM) receptor interaction, nuclear factor κB (NF-κB) signaling, tumor necrosis factor (TNF) signaling, osteoclast differentiation, mammalian target of rapamycin (mTOR) signaling, and mitogen-activated protein kinase (MAPK) signaling. Thus, we identified subsets of mRNAs that are differentially regulated during the process of OLF by applying a systematic array approach.

Identification of Differentially Expressed lncRNAs in OLF

To investigate the differentially expressed lncRNAs during the process of OLF, we performed lncRNA expression profiles in 4 OLF and 4 normal LF tissues, and hierarchical clustering (Figure 2A) showed distinguishable lncRNA expression patterns among samples.

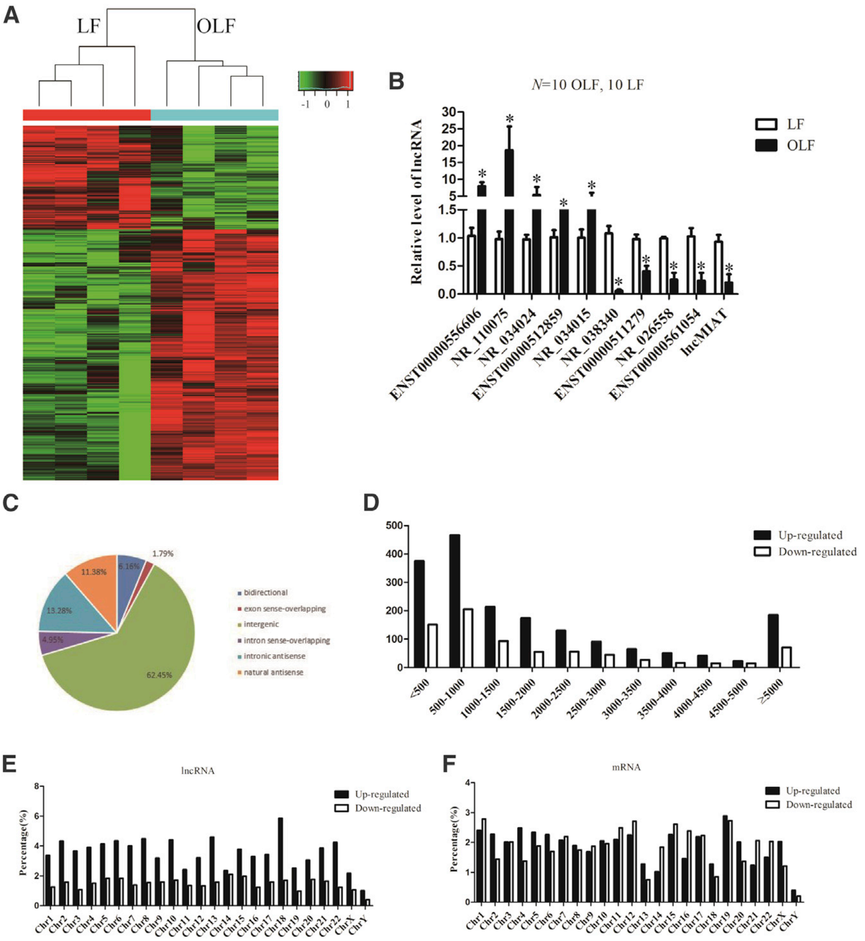

Figure 2. The Differentially Expressed lncRNAs in OLF

(A) Microarray analysis for lncRNAs was performed with RNA extracted from ossification (n = 4) and normal (n = 4) *ligamentum flavum*. Hierarchical cluster analysis of significantly differentially expressed lncRNAs: bright green, under-expression; gray, no change; bright red, overexpression. (B) Differential expression of ten representative lncRNAs was validated in human ossified and normal *ligamentum flavum* tissues by qPCR (n = 10 per group). GAPDH was used as an internal control. (C) The deregulated lncRNAs were divided into intergenic, antisense, natural antisense, bidirectional, intron sense-overlapping, and exon sense-overlapping. (D) The deregulated lncRNAs were classified according to different lengths. (E and F) The deregulated lncRNAs (E) and mRNAs (F) were classified according to their percentages in different chromosomes. Data are presented as mean ± SEM; p values were analyzed by Student's t test; $^*p < 0.05$.

From the lncRNA expression profiling data, 2,567 lncRNAs were found to be differentially expressed in OLF tissues (fold change > 2, $p < 0.05$), of which 1,817 lncRNAs were upregulated and 750 lncRNAs were downregulated. The number of upregulated lncRNAs is much higher than that of the downregulated ones, suggesting that this is like a ligament-to-bone conversion. Subsequently, we randomly selected five upregulated and five downregulated lncRNAs to perform qRT-PCR to verify the microarray results in extension samples of OLF tissues and normal LF tissues. The results showed that the deregulated lncRNAs are consistent with the microarray data (Figure 2B; Table S3). Further lncRNAs subgroup analysis showed that the majority of differentially expressed lncRNAs in this study were intergenic (62.45%), and others include intronic antisense (13.28%), natural antisense (11.38%), bidirectional (6.16%), intron sense-overlapping (4.95%), and exon sense-overlapping (1.79%) (Figure 2C). Character analysis showed that the differentially expressed lncRNAs are mainly between 200 and 1,000 bp in length (Figure 2D). It has been reported that chromosomal imbalances are associated with various diseases.[18] In this study, aberrantly expressed lncRNAs and mRNAs were found to be most prone to be located on chromosomes 18 and 19, respectively, after normalization with the amount of genes located on each chromosome (Figures 2E and 2F). Thus, we identified a series of lncRNAs that are differentially expressed between OLF and normal LF tissues.

To further confirm that the deregulated lncRNAs function in the process of ossification, we selected two upregulated lncRNAs, ENST00000608133 and ENST00000599584, in OLF (Table S3). The transcripts of these two lncRNAs were upregulated in OLF and in human bone marrow mesenchymal stem cells (hMSCs) under osteogenic induction (Figures 3A–3C). Knockdown of these lncRNAs with the respective small interfering RNA (siRNA) reduced the mRNA levels of alkaline phosphatase (ALP), COL1α1, BGLAP, and RUNX2, all of which are osteogenic differentiation-associated genes, and ALP staining during the process of hMSC differentiation into osteoblasts (Figures 3D–3F). These data indicate that the deregulated lncRNAs can regulate ossification-associated genes.

Construction of the lncRNA-mRNA Co-expression Network

To date, the functions of most lncRNAs have not been annotated, and the functional forecast of lncRNAs is made according to the annotations of the co-expressed mRNA function. Therefore, we selected ten differentially expressed mRNAs (RUNX2, MMP9, BGLAP, IL6, COL2α1, AKT1, TNFRSF1A, TGFB2, BMP4, and PIK3R3) that have been reported to be closely associated with ossification and osteogenic differentiation[7,19–26] together with co-expressed lncRNAs in the microarray based on the degree of correlation to build a coding-non-coding (CNC) network to predict the functions of the deregulated lncRNAs (Figure S2). Our data showed that the co-expression network was composed of 704 network nodes and 748 connections among ten coding genes and lncRNAs. The

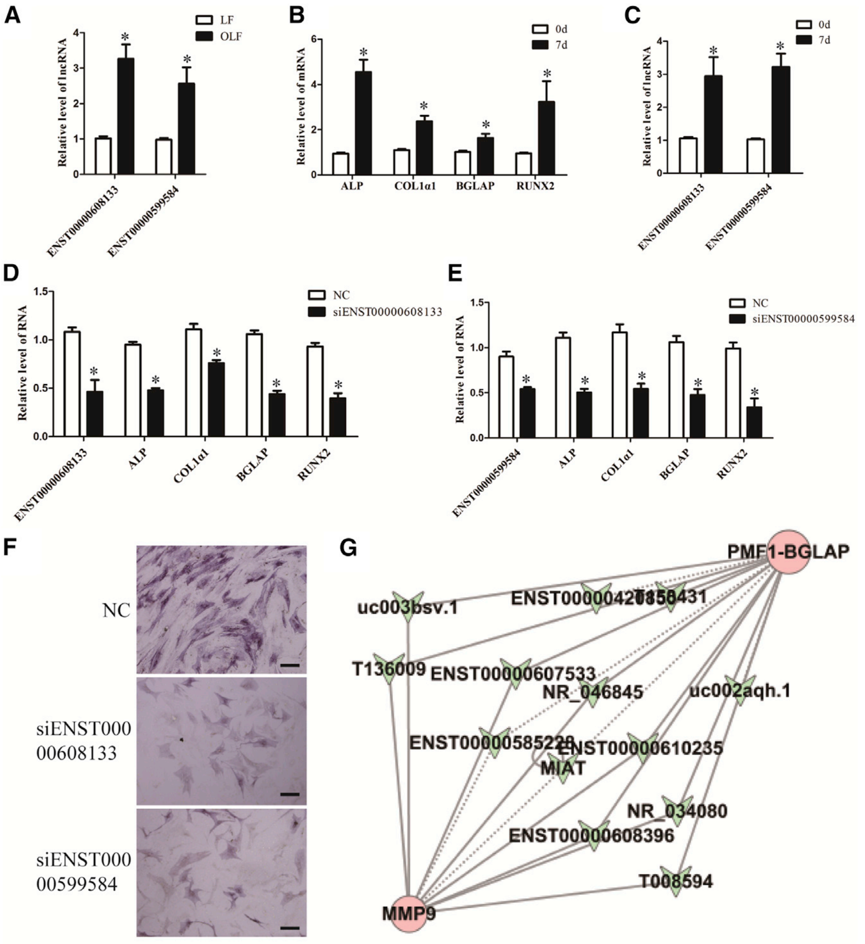

Figure 3. The Function of Two Upregulated lncRNAs and Co-expression Network of MMP9 and BGLAP with Their Associated lncRNAs

(A) The differential expression of ENST00000608133 and ENST00000599584 was validated with total RNA isolated from OLF and normal LF by qPCR (n = 10 per group). GAPDH was used as an internal control. (B and C) qPCR analysis of ALP, COL1α1, BGLAP, and RUNX2 (B) and ENST00000608133 and ENST00000599584 (C) was performed, with total RNA isolated from hMSCs and treated with osteogenic medium for 0 and 7 days. (D and E) hMSCs were transfected with siENST00000608133 (D), siENST00000599584 (E), or the negative control and further cultured in osteogenic medium for 7 days. The RNA levels of ENST00000608133 (D), ENST00000599584 (E) ALP, Col1α1, BGLAP, and RUNX2 were detected by qPCR. (F) Representative images of ALP staining of hMSCs after transfection with siENST00000608133, siENST00000599584, or the negative controls and further culture in osteogenic medium for 7 days. Scale bars, 400 μm. (G) Co-expression network of MMP9 and BGLAP with their associated lncRNAs. The network is based on PCC. V nodes represent lncRNA, and circle nodes represent mRNAs. Solid lines represent a positive relationship, and dashed lines represent a negative relationship. Data are presented as mean ± SEM for at least triplicate experiments. The p values were analyzed by Student's t test; $^*p < 0.05$.

co-expression network could imply that these lncRNAs are involved in the process of OLF. For instance, the sub-network of MMP9 and BGLAP showed that the downregulated lncRNA MIAT was negatively correlated with these mRNAs (Figure 3G), which is consistent with a study showing that the lncRNA MIAT inhibits osteogenic differentiation.[27] Therefore, the CNC network could predict the functions of co-expressed lncRNAs in OLF.

Identification of Differentially Expressed miRNAs in OLF

To identify the miRNA expression profiles in OLF, we performed miRNA sequencing, and the results showed that 12 upregulated and 16 downregulated miRNAs are identified in OLF (fold change > 2, $p < 0.05$) (Figure 4A). Subsequently, qPCR confirmed differential expression of these miRNAs between OLF and LF tissues (Figure 4B; Table S4). Moreover, the differentially expressed miRNAs were found to be prone to be located on chromosomes 1, 9, 14, and X (Figure 4C).

To further confirm the function of the deregulated miRNAs, we selected a downregulated miRNA, miR-19b-3p, from miRNA sequencing (Table S4). miR-19b-3p was downregulated in OLF and in hMSCs under osteogenic induction (Figures 4D and 4E). Overexpression of miR-19b-3p with mimics reduced the mRNA levels of the osteogenic differentiation-associated genes and ALP staining during the process of osteogenic differentiation (Figures 4F and 4G). These results indicate that miR-19b-3p regulates ossification-related genes.

Construction of the Target Prediction Network of miRNA-mRNA

To identify the function of the aberrantly expressed miRNAs, the prediction of miRNA target genes was performed by the TargetScan, miRBase, and miRanda databases, and subsequently overlapped these databases. All of these predicted 1,201 mRNAs were used to perform the GO and KEGG pathway analysis (Figure S3A). However, the top ten signaling pathways, including the transforming growth factor β (TGF-β) signaling pathway, Wnt signaling pathway, and Hippo signaling pathway, are obviously associated with ossification (Figure S3B). To find a much more specific miRNA-mRNA interaction network, we used the differentially expressed mRNAs identified previously (Figure 1A) to overlap with the 1,201 predicted targets, and only genes that negatively correlated with their corresponding miRNAs were included (Figure S4). As shown in Figures 5A and 5B, the GO analysis showed that the differentially expressed miRNAs targets were mainly associated with skeletal system development, ECM, endochondral ossification, replacement ossification, and Wnt-activated receptor

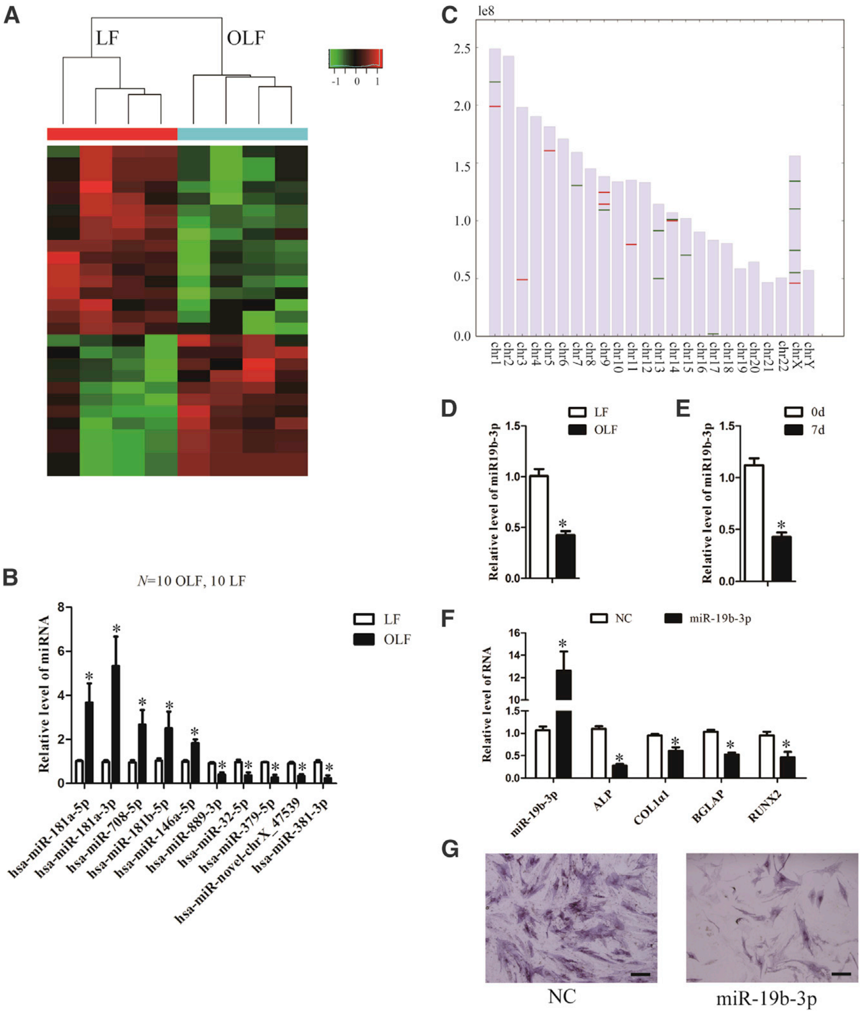

Figure 4. Differentially Expressed miRNAs in OLF and the Function of miR-19b-3p

(A) Sequencing analysis for miRNA was performed with RNA extracted from ossification (n = 4) and normal (n = 4) LF. Hierarchical cluster analysis of significantly differentially expressed lncRNAs: bright green, under-expression; gray, no change; bright red, overexpression. (B) Differential expression of ten representative miRNAs was validated in human ossified and normal LF tissues by qPCR (n = 10 per group). (C) The differentially expressed miRNAs were distributed on different chromosomes. (D and E) qPCR analysis of miR-19b-3p was performed with total RNA isolated from ossified and normal LF (D) and hMSCs treated with osteogenic medium for 0 and 7 days (E). (F) hMSCs were transfected with miR-19b-3p mimic or the negative control and further cultured in osteogenic medium for 7 days. The RNA levels of miR-19b-3p, ALP, Col1α1, BGLAP, and RUNX2 were detected by qPCR. U6 was used as an internal control for miR-19b-3p. GAPDH was used as an internal control for mRNA. (G) Representative images of ALP staining of hMSCs after transfection with miR-19b-3p mimic or the negative controls and further culture in osteogenic medium for 7 days. Scale bars, 400 μm. Data are presented as mean ± SEM for at least triplicate experiments. The p values were analyzed by Student's t test; *$p < 0.05$.

activity. Furthermore, KEGG pathway analysis also revealed that the upregulated mRNAs are associated with the signaling pathways of ossification and the downregulated mRNAs are associated with osteoclast differentiation, indicating that differentiation to osteoclasts is repressed in the process of ossification (Figures 6A and 6B). Taken together, we have identified subsets of miRNAs that are differentially regulated in OLF, and the miRNA-mRNA network implies that these miRNAs play a core role in the transcription-regulatory network during the process of OLF.

Identification of Differentially Expressed circRNAs in OLF

Arraystar human circRNA array analysis was also adopted for profiling human circRNA expression. The data showed that 244 circRNAs were upregulated and 383 circRNAs were downregulated in OLF tissues (fold change > 2, $p < 0.05$) (Figure 7A). The expression of ten randomly selected circRNAs was validated by qPCR, which was consistent with the results of the microarray (Figure 7B; Table S5).

It has been reported that circRNAs can function through their parental genes. Therefore, a correlation between the differentially expressed circRNAs and their linear counterparts was constructed, followed by GO and KEGG pathway analyses. However, the results of the GO and KEGG analyses showed that the top ten GO items and pathways are not obviously associated with ossification, apart from the mTOR signaling pathway (Figures 7C, 7D, S5A, and S5B). These data indicate that circRNA deregulation might be a side effect of the massive deregulation observed in OLF. To investigate whether the deregulated circRNAs, which might be a side effect of massive deregulation, are involved in OLF, we selected an upregulated circRNA, circ_0050139, which is a target of miR-19b-3p (Table S5). This molecule was confirmed to be overexpressed in OLF and hMSCs under osteogenic induction (Figures 7E and 7F). We further silenced it by siRNA to detect whether the expression of osteogenic differentiation-associated genes was affected. The results showed that knockdown of circRNA_0050139 reduced the transcripts of ALP, COL1α1, BGLAP, and RUNX2 and ALP staining in hMSCs under osteogenic induction (Figures 7G and 7H), confirming that this deregulated circRNA is involved in ossification.

Construction of the miRNA-lncRNA-circRNA-mRNA network

Furthermore, we predicted miRNAs that target the differentially expressed circRNAs and overlapped them with the deregulated

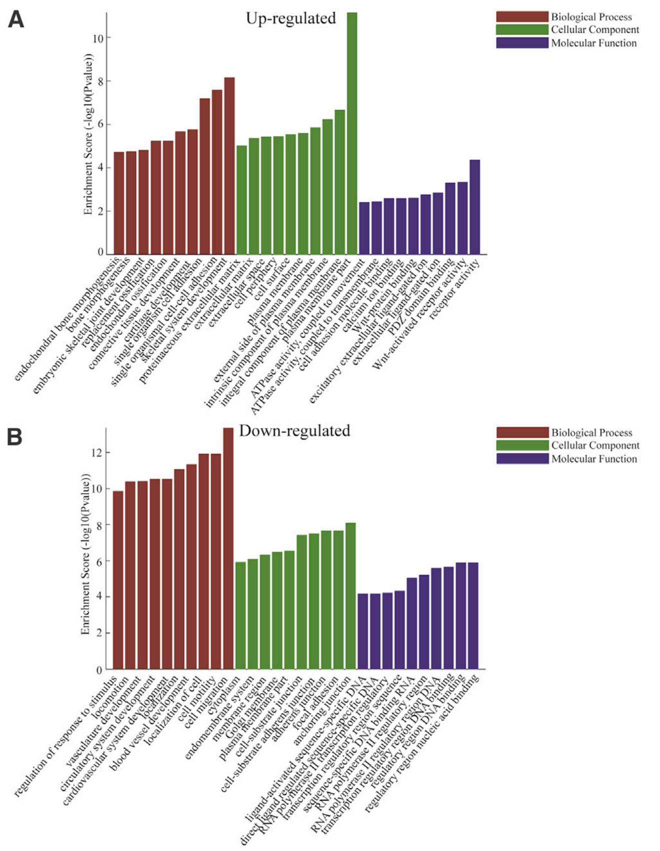

Figure 5. GO Analysis of the MicroRNA-mRNA Network

(A and B) GO annotation of the linear counterparts of upregulated (A) and downregulated (B) mRNAs with the top ten enrichment score covering domains of biological processes, cellular components, and molecular functions was performed. Both up- and downregulated mRNAs are significantly changed, with fold change > 2.0, p < 0.05 between OLF and normal LF tissues.

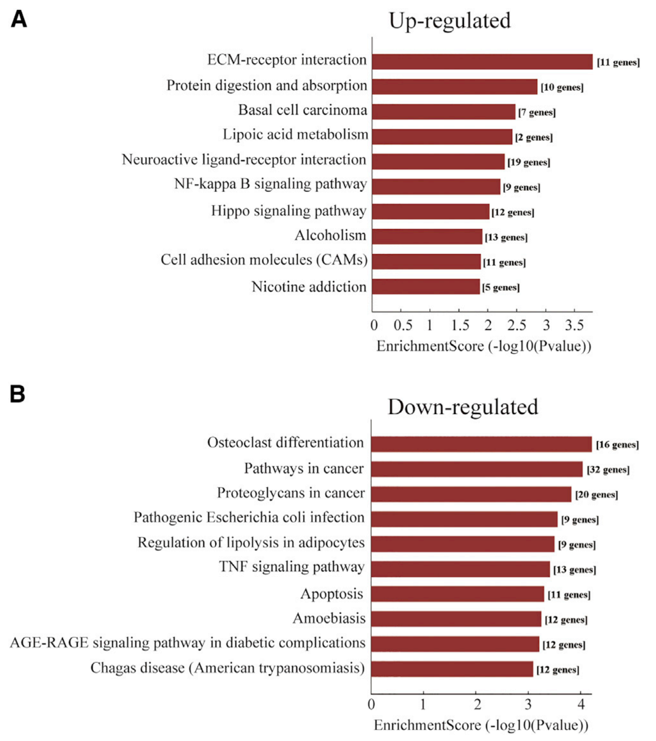

Figure 6. KEGG Analysis of the MicroRNA-mRNA network.

(A and B) KEGG pathway enrichment analysis of upregulated (A) and downregulated (B) mRNAs with the top ten enrichment scores was performed. Both up- and downregulated mRNAs are significantly changed, with fold change > 2.0, p < 0.05 between OLF and normal LF tissues.

miRNAs from the sequencing data to subsequently construct a circRNA-miRNA-mRNA relationship network, including the overlapped miRNAs, matching circRNA- and miRNA-targeted mRNAs (Figure 8A). The results showed that the miRNAs in this network, such as miR-181a, which has been reported to promote osteoblastic differentiation through repression of TGF-β signaling,[28] and the mRNAs in this network, including LPP, ATF7, and ACTA1, are involved in the process of OLF.[29–31]

Given the evidence that the deregulated lncRNAs ENST00000608133 and ENST00000599584, which were actually targets of miR-19b-3p, are related to ossification by regulation, at least, of RUNX2, which is also regulated by miR-19b-3p and circ_0050139, we conclude that the deregulated miRNAs, lncRNAs, and circRNAs regulate ossification-associated genes (Figure 8B). Furthermore, we confirmed the co-regulation with experiments showing that overexpression of miR-19b-3p downregulates the levels of ENST00000608133, ENST00000599584, and circ_0050139 (Figure 8C).

DISCUSSION

Various cytokines, growth factors, and miRNAs have been shown to regulate the genes that orchestrate proliferation and differentiation in the process of ossification;[32,33] however, little information exists regarding the lncRNAs and circRNAs that regulate osteogenesis differentiation. In this study, we found that some mRNAs, lncRNAs, miRNAs, and circRNAs are altered during the OLF ossification process.

Serine peptidases (proteases) (for instance, human HTRA1 [high-temperature requirement serine protease A1]) positively regulate osteogenesis and mineralization of differentiating bone-forming cells through the modulation of ECM protein and have been implicated in musculoskeletal development.[34] Figure 1C shows that the highest enrichment of upregulated genes concerning molecular function is associated with serine peptidase activity, which indicates that, in the process of ossification, serine peptidases promote bone formation. This result was linked with the rest of the data and the ossification process. Blood supply is essential for the function of ligaments, and angiogenesis is regulated by genes. Our chromatin immunoprecipitation (ChIP) data showed that the angiogenesis genes are downregulated (Figure S1A) (for instance, VEGF), indicating that reduced angiogenesis and the subsequent decreased blood supply in ligaments,

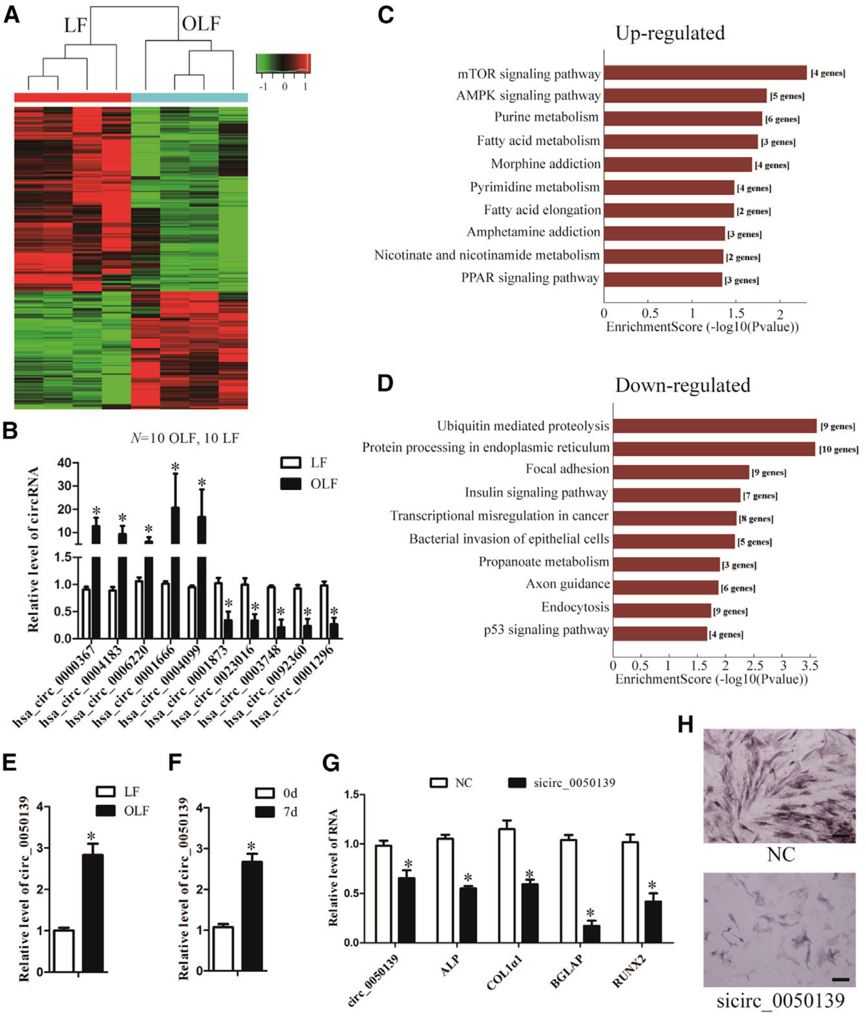

Figure 7. Differently Expressed circRNAs in OLF and the Function of circ_0050139

(A) Microarray analysis for circRNA was performed with RNA extracted from ossification (n = 4) and normal (n = 4) *ligamentum flavum*. Hierarchical cluster analysis of significantly differentially expressed circRNAs: bright green, under-expression; gray, no change; bright red, overexpression. (B) Differential expression of ten representative circRNAs was validated in human ossified and normal LF tissues by qPCR (n = 10 per group). (C and D) KEGG pathway enrichment analysis of linear counterparts of the upregulated (C) and downregulated (D) circRNAs with the top ten enrichment scores was performed. Both up- and downregulated circRNAs are significantly changed, with fold change > 2.0, p < 0.05 between OLF and normal LF tissues. (E and F) qPCR analysis of circ_0050139 was performed with total RNA isolated from ossified and normal LF (E) and hMSCs treated with osteogenic medium for 0 and 7 days (F). (G) hMSCs were transfected with sicirc_0050139 or the negative control and further cultured in osteogenic medium for 7 days. The RNA levels of circ_0050139, ALP, Col1α1, BGLAP, and RUNX2 were detected by qPCR. GAPDH was used as an internal control. (H) Representative images of ALP staining of hMSCs after transfection with sicirc_0050139 or the negative controls and further culture in osteogenic medium for 7 days. Scale bars, 400 μm. Data are presented as mean ± SEM; p values were analyzed by Student's t test; *p < 0.05.

but not in bone tissue, might cause ossification. On the other hand, the enrichment of blood vessels in normal ligaments might be more than that in ossified ligaments, with which the ChIP analysis was performed, and it showed that the angiogenesis genes are downregulated. We will conduct research to investigate this in a future study. In addition, very few genes have been identified and confirmed to be directly related to ligament function. This is also the reason why we have found an upregulation of bone-related genes but no downregulation of ligament-related genes.

The ossification of ligament tissue is a complex process characterized by gradually increasing osteophytes and regulated by signaling-regulatory networks, including growth factors, transcription factors, and ncRNAs, a number of which form feedback loops controlling the process of osteogenic differentiation.[35] For instance, activated Wnt signaling through treatment with Wnt3a induces TAZ expression and increases its nuclear localization to stimulate osteogenic differentiation.[36] Activated mTOR signaling promotes expression of the transcription factor RUNX2 to induce osteogenesis.[37] TNF-α-activated NF-κB signaling increases the level of miR-150-3p, which directly targets the 3′ UTR of β-catenin mRNA and, in turn, represses its expression to inhibit the osteogenic differentiation.[38] It has also been reported that there exists a feedback-regulatory loop consisting of miR-26a, GSK3β, and CCAAT/enhancer binding protein alpha (C/EBPα) that regulates osteogenesis.[39]

To date, the transcription profiling of OLF was mainly associated with genes encoding proteins, and much less was known about ncRNAs. Recent reports have demonstrated that ncRNAs participate in modulating numerous biological functions and pathological processes through regulating gene expression at the transcriptional and post-transcriptional levels.[40,41] For instance, a recent study has reported that the liver-enriched lnc-LFAR1 promotes hepatic stellate cell activation and TGFβ-induced hepatocyte apoptosis *in vitro* and aggravates both CCl_4- and bile duct ligation-induced liver fibrosis by activating the TGF-β and Notch pathways.[12] Also, it has been demonstrated that ciRS-7 can repress Alzheimer's disease (AD) development by suppressing NF-κB protein synthesis and inducing its cytoplasmic localization, promoting UCHL1 expression and UCHL1-induced amyloid precursor protein (APP) and BACE1 ubiquitination and degradation.[13] Moreover, primary ligament cells were preferentially used for transcription profiling in most OLF

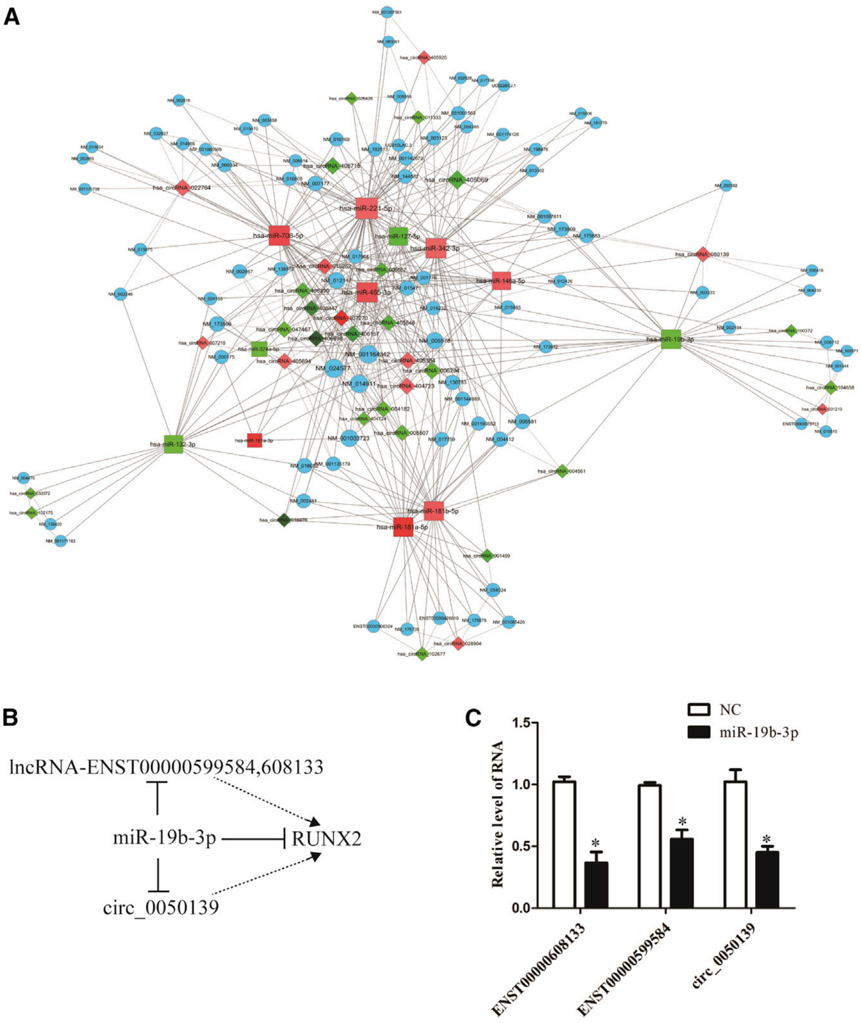

Figure 8. Integrated circRNA-microRNA-mRNA ceRNA Network Analysis

(A) The competing endogenous RNA network has been based on circRNA-miRNA and miRNA-mRNA interactions. In this network, the circRNAs connect expression-correlated mRNAs via miRNAs. Square nodes represent miRNAs, diamonds represent circRNAs, and blue circles represent mRNAs. Red and green colors represent up- and downregulation, respectively. The shade of darkness represents fold change. The size of circles represents p values, with larger size owing smaller p value. Solid lines represent directed relationships, and dashed lines represent undirected relationships. (B) A simplified scheme was constructed, including the upregulated ENST00000608133, ENST00000599584, circ_0050139 and RUNX2 and the downregulated miR-19b-3p. (C) hMSCs were transfected with miR-19b-3p mimic or the negative control, and the RNA levels of ENST00000608133, ENST00000599584, and circ_0050139 were detected by qPCR. GAPDH was used as an internal control. Data are presented as mean ± SEM for at least triplicate experiments. The p values were analyzed by Student's t test; *p < 0.05.

research.[42,43] In this study, for the first time, we used normal and ossified ligament tissues for transcription profiling, which reflect more closely the change in expression of ncRNAs and mRNA of OLF *in vivo*.

Despite their poor conservation and low levels of expression compared with protein-coding genes, lncRNAs are often regulated by transcription factors and are expressed in a cell- or tissue-specific manner.[12,44] In this study, 1,817 lncRNAs were upregulated and 750 lncRNAs were downregulated, suggesting that lncRNAs are more likely to be induced during the process of OLF. Further lncRNA subgroup analysis showed that the majority of differentially expressed lncRNAs were intergenic, which is consistent with the distribution of lncRNAs. In addition, from the lncRNA-mRNA co-expression network, we found that the mRNAs of MMP9 and BGLAP co-expressed with multiple lncRNAs, forming a complex network, which showed that a downregulated lncRNA, MIAT, was negatively correlated with the mRNAs of MMP9 and BGLAP, consistent with a previous study showing that the lncRNA MIAT inhibits osteogenic differentiation.[27] In our study, most of the lncRNAs in the co-expression network were not yet annotated. It is very much worth it to perform further studies to reveal the underlying mechanisms of these lncRNAs.

circRNAs are drawing increased attention as one of the top hot ncRNAs. Evidence is emerging that circRNAs can participate in the regulation of gene expression in various ways. It has been reported that circRNAs can function through their parental genes. For instance, circular RNA that spans several exons of Ubiquitin (Ub) protein ligase 3 (E3) (cir-ITCH) can regulate the Wnt pathway by increasing the level of ITCH in esophageal squamous cell carcinoma.[45] Additionally, many more circRNAs have been reported to harbor multiple miRNA binding sites, which seem to be a typical feature of this class of RNA molecules; this suggests that circRNA can act as a sponge of miRNA to regulate target genes. To date, various studies have made substantial progress regarding the function of CDR1 natural antisense transcript (NAT) to act as an miR-7 sponge *in vitro* and *in vivo* and, thus, have termed this circular transcript ciRS-7.[46,47] Therefore, in this study, a correlation between the differentially expressed circRNAs and their linear counterparts was constructed, followed by GO and KEGG pathway analyses. The results of the GO and KEGG analyses showed that the circRNA-correlated linear counterparts are not obviously associated with ossification, apart from the mTOR and peroxisome proliferators-activated receptor (PPAR) signaling pathways, indicating that the deregulated circRNAs might also be a side effect of the massive gene expression deregulation observed in OLF. However, in this study, as shown by the evidence

of the selected circ_0050139, which regulates osteogenic differentiation-associated genes, it suggests that the differentially expressed circRNAs, a side effect of massive deregulation, may function by sponging the matched miRNA, thus releasing the miRNA inhibition instead of binding to their parental genes during the process of OLF.

In conclusion, our findings provide, for the first time, a systematic perspective on the potential function of ncRNAs and mRNAs during the process of OLF, illuminating the potential mechanism of OLF pathogenesis and providing new targets for OLF.

MATERIALS AND METHODS

Sample Collection

Ossified and normal OLF tissues were obtained from OLF or spinal trauma patients who underwent posterior decompression laminectomy. We confirmed the diagnosis of OLF and spinal trauma by X-ray, computed tomography (CT), and MRI before the operation. The specimens were collected during surgery, rinsed with PBS, frozen in liquid nitrogen, and stored at −80°C for further study. Clinical samples were obtained after receiving informed consent from the patients. The study methodologies conformed to the standards set by the Declaration of Helsinki. This study was approved by the ethical review committee of Tianjin Medical University General Hospital.

RNA Extraction and Quality Control

To isolate total RNA from the tissue, the frozen tissue was ground in liquid nitrogen, and total RNA was extracted with Trizol reagent (Life Technologies). Total RNA was extracted from cells with Trizol reagent. RNA quantity and quality were measured by NanoDrop ND-1000. RNA integrity was assessed by standard denaturing agarose gel electrophoresis and an Agilent 2100 Bioanalyzer. All RNA samples were stored at −80°C until further processing.

Microarray Analysis

Arraystar Human lncRNA Microarray V4.0 was adopted for detection of lncRNAs and mRNA expression. Arraystar Human circRNA Array V2 (8x15K, Arraystar) was adopted for profiling circRNAs expression. All microarray analyses were performed by KangChen Bio-tech (Shanghai, China). Briefly, sample labeling and array hybridization were performed according to the Agilent One-Color Microarray-Based Gene Expression Analysis protocol (Agilent Technology) with minor modifications. The mRNA was purified from total RNA after removal of rRNA (mRNA-ONLY Eukaryotic mRNA Isolation Kit, Epicenter). Each sample was then amplified and transcribed into fluorescent cRNA along the entire length of the transcripts without 3′ bias utilizing a random priming method (Arraystar Flash RNA Labeling Kit, Arraystar). The labeled cRNAs were purified by RNeasy Mini Kit (QIAGEN). The concentration and specific activity of the labeled cRNAs (picomoles Cy3 per microgram cRNA) were measured by NanoDrop ND-1000. 1 μg of each labeled cRNA was fragmented by adding 5 μL 10× blocking agent and 1 μL of 25× fragmentation buffer, and then the mixture was heated at 60°C for 30 min. Finally, 25 μL 2× GE Healthcare hybridization buffer was added to dilute the labeled cRNA, and 50 μL of hybridization solution was dispensed into the gasket slide and assembled on the lncRNA expression microarray slide. The slides were incubated for 17 hr at 65°C in an Agilent hybridization oven. The hybridized arrays were washed, fixed, and scanned using the Agilent DNA microarray scanner (part number G2505C). Agilent Feature Extraction software (version 11.0.1.1) was used to analyze the acquired array images. Quantile normalization and subsequent data processing of lncRNA and mRNA were performed using the GeneSpring GX v12.1 software package (Agilent Technologies). A series of data processing, including quantile normalization of circRNA, was performed using the R software limma package. Differentially expressed lncRNAs, mRNAs, and circRNAs between the two groups were identified through fold change and p value and false discovery rate (FDR) filtering. Hierarchical clustering was performed to generate an overview of the characteristics of expression profiles based on values of significant differentially expressed transcripts.

miRNA Sequence

Illumina NextSeq 500 was used for library construction following the manufacturer's protocol. It included the following steps: 3′ adaptor ligation, 5′ adaptor ligation, cDNA synthesis, PCR amplification, and size selection of ∼135- to 155-bp PCR-amplified fragments (corresponding to ∼15- to 35-nt small RNAs). The libraries were denatured as single-stranded DNA molecules, captured on Illumina flow cells, amplified *in situ* as clusters, and finally sequenced for 51 cycles on Illumina NextSeq according to the manufacturer's instructions. All profiling work was done with the help of KangChen Bio-tech (Shanghai, China). After sequencing, the Solexa CHASTITY quality filtered reads were harvested as clean reads. The adaptor sequences were trimmed, and the adaptor-trimmed reads (≥15 nt) were left. miRDeep2 software was used to predict the novel miRNAs with these trimmed reads. Then the trimmed reads were aligned to merged pre-miRNA databases (known pre-miRNA from miRBase v21 plus the newly predicted pre-miRNAs) using Novoalign software (v2.07.11) with, at most, one mismatch. We used the most abundant isomiR, the mature miRNA annotated in miRBase and all isoforms of miRNA (5p or 3p) to calculate miRNA expression. When comparing the differentially expressed miRNA profiles between two groups, the fold change and p value and FDR were calculated and used to identify significant differentially expressed miRNAs. Hierarchical clustering was performed to generate an overview of the characteristics of expression profiles based on values of significant differentially expressed transcripts.

Correlation and Co-expression Analysis

The co-expression analysis was based on calculating the Pearson correlation coefficient (PCC) between coding genes and noncoding transcripts according to their expression levels. The absolute values of parameter PCC $\geq$ 0.968 and $p < 0.05$ were recommended and retained for further analysis.

ceRNA Network Analysis

circRNAs-miRNAs and miRNAs-mRNAs whose expression levels shared a meaningful correlation were subjected to the analysis. The

potential miRNA response elements were searched on the sequences of circRNAs and mRNAs, and the overlap of the same miRNA seed sequence binding site both on the circRNAs and the mRNA predicted circRNA-miRNA-mRNA interaction.

GO and KEGG Pathway Analysis

GO analysis was conducted to construct meaningful annotation of genes and gene products in a wide variety of organisms. The ontology has covered domains of biological processes (BPs), cellular components (CCs), and molecular functions (MFs). The-log10 (p value) denotes the enrichment score, representing the significance of GO term enrichment among differentially expressed genes. KEGG pathway analysis was also performed to harvest pathway clusters covering our knowledge of the molecular interaction and reaction networks in differentially regulated gene profiling. The −log10 (p value) denotes the enrichment score, showing the significance of the pathway correlations. The p value was corrected by FDR.

Real-Time qPCR

Total RNA was extracted from OLF tissues with Trizol reagent (Life Technologies, Grand Island, NY, USA). For qPCR of mRNA, lncRNA, and circRNA, total RNA was digested with DNase I (Takara, Dalian, China). Briefly, the 10-μL reverse transcription (RT) reaction (1 μg RNA, 1 μL buffer, 1 μL DNase1, and water) was incubated for 15 min at 37°C, followed by addition of 1 μL of EDTA, incubation for 10 min at 65°C, and then maintenance at 4°C. Next, the first-strand cDNA was synthesized using avian myeloblastosis virus (AMV) reverse transcriptase (Thermo Fisher Scientific, Basingstoke, UK) according to the manufacturer's instructions. For real-time PCR, all reactions were performed in triplicate with SYBR Green Master Mix (Takara, Dalian, China) under the following conditions: 15 min at 95°C for initial denaturation, followed by 40 cycles of segments of 95°C for 30 s and 60°C for 30 s in the Light Cycler 96 real-time PCR system (Roche, Mannheim, Germany). The expression levels of the housekeeping gene GAPDH were used to normalize the expression levels of the genes of interest. For qPCR of miRNA, reverse transcribed using The First-strand cDNA Synthesize (Sangon, B532451). For real-time PCR, all reactions were performed in triplicate with SYBR Green Master Mix (Takara, Dalian, China) under the following conditions: 15 min at 95°C for initial denaturation, followed by 40 cycles of segments of 95°C for 30 s and 60°C for 30 s in the Light Cycler 96 real-time PCR system (Roche, Mannheim, Germany). The expression levels of U6 were used to normalize the expression levels of the genes of interest. The primers used in the real-time PCR are listed in Table S6.

Cell Cultures and Treatment

The primary hMSCs (Cyagen Biosciences) were cultured in alpha modified Eagle's medium (Invitrogen) supplemented with 1% penicillin and streptomycin (Gibco) and 10% fetal bovine serum (FBS, Gibco). Cells were cultured under conditions of 5% CO_2. Confluent cells were digested with 0.25% trypsin including 10 mM EDTA, resuspended in corresponding medium, and plated into plates with a suitable density. To induce osteoblast differentiation, 50 μg/mL of ascorbic acid (Sigma), 5 mM β-glycerophosphate (Sigma), and 10 nM dexamethasone (Sigma) were add to the culture medium.

Transfection Assay

hMSCs were cultured overnight until 50% confluence and then transfected with siRNA (supplied by GenePharma) using Lipofectamine 2000 (Invitrogen) according to the instructions. After 4 hr of transfection, the medium containing transfection reagent was replaced by fresh induction medium. After culturing for an additional 48 hr, cells were used for further experiments. The siRNA sequences for lncRNAs and circRNA are listed in Table S6.

Alkaline Phosphatase Staining

Alkaline phosphatase staining was monitored with the 5-bromo-4-chloro-3-indolyl phosphate (BCIP) and NBT Alkaline Phosphatase Color Development Kit (C3206, Beyotime Institute of Biotechnology) in accordance with the instructions. Briefly, cells were fixed by immersion in 4% paraformaldehyde at 4°C for 30 min and rinsed in PBS for 5 min. The alkaline phosphatase stain was then added to the plates for 30 min while protecting the plates from light. The pictures were monitored by microscopy after rinsing in deionized water for 5 min.

Statistical Analysis

Data are expressed as mean ± SEM. All the statistical analyses were performed with SPSS 13.0 (IBM, Armonk, NY, USA). Statistical analysis was performed using either Student's t test (two-group comparison) or one-way analysis of variance (more than two groups), followed by *post hoc* comparison, and differences with $p < 0.05$ were considered significant.

Accession Numbers

The accession numbers for the lncRNA and mRNA microarray, circRNA microarray, and miRNA-seq data reported in this paper are NCBI GEO: GSE106253, GSE106255, and GSE106256.

SUPPLEMENTAL INFORMATION

Supplemental Information includes five figures and six tables and can be found with this article online at https://doi.org/10.1016/j.omtn.2018.07.018.

AUTHOR CONTRIBUTIONS

W.H. and Y.X. conceived and designed the study. Y. Han, Y. Hong, and L.L. performed the majority of the experiments. Z.S., X.H., and T.C. gave technical support and conceptual advice. W.H., Y. Han, and K.Z. analyzed the data and wrote the manuscript. L.L., T.L., H.X., and Y.T. collected clinical samples. Y. Han, Y. Hong, K.Z., Z.Z., J.W., Q.L., M.Z., Y.X., and W.H. contributed reagents, materials, and analysis tools.

CONFLICTS OF INTEREST

We declare no competing interests.

ACKNOWLEDGMENTS

This work was supported by the National Natural Science Foundation of China (81471403, 81871124, 81670558, and 81800542) and the Postgraduate Innovation Fund of "13th Five-Year Comprehensive Investment," Tianjin Medical University (YJSCX201702).

REFERENCES

1. Guo, J.J., Luk, K.D., Karppinen, J., Yang, H., and Cheung, K.M. (2010). Prevalence, distribution, and morphology of ossification of the ligamentum flavum: a population study of one thousand seven hundred thirty-six magnetic resonance imaging scans. Spine *35*, 51–56.
2. Lang, N., Yuan, H.S., Wang, H.L., Liao, J., Li, M., Guo, F.X., and Chen, Z.Q. (2013). Epidemiological survey of ossification of the ligamentum flavum in thoracic spine: CT imaging observation of 993 cases. Eur. Spine J. *22*, 857–862.
3. Mobbs, R.J., and Dvorak, M. (2007). Ossification of the ligamentum flavum: diet and genetics. J. Clin. Neurosci. *14*, 703–705.
4. Fan, D., Chen, Z., Chen, Y., and Shang, Y. (2007). Mechanistic roles of leptin in osteogenic stimulation in thoracic ligament flavum cells. J. Biol. Chem. *282*, 29958–29966.
5. Uchida, K., Yayama, T., Cai, H.X., Nakajima, H., Sugita, D., Guerrero, A.R., Kobayashi, S., Yoshida, A., Chen, K.B., and Baba, H. (2011). Ossification process involving the human thoracic ligamentum flavum: role of transcription factors. Arthritis Res. Ther. *13*, R144.
6. Lu, Y., Ding, M., Li, N., Wang, Q., Li, J., Li, X., Gu, J., Im, H.J., Lei, G., and Zheng, Q. (2014). Col10a1-Runx2 transgenic mice with delayed chondrocyte maturation are less susceptible to developing osteoarthritis. Am. J. Transl. Res. *6*, 736–745.
7. Chen, H., Ghori-Javed, F.Y., Rashid, H., Adhami, M.D., Serra, R., Gutierrez, S.E., and Javed, A. (2014). Runx2 regulates endochondral ossification through control of chondrocyte proliferation and differentiation. J. Bone Miner. Res. *29*, 2653–2665.
8. Consortium, E.P.; ENCODE Project Consortium (2012). An integrated encyclopedia of DNA elements in the human genome. Nature *489*, 57–74.
9. Guil, S., and Esteller, M. (2015). RNA-RNA interactions in gene regulation: the coding and noncoding players. Trends Biochem. Sci. *40*, 248–256.
10. Lian, Y., Yan, C., Xu, H., Yang, J., Yu, Y., Zhou, J., Shi, Y., Ren, J., Ji, G., and Wang, K. (2018). A novel lncRNA, LINC00460 affects cell proliferation and apoptosis by regulating KLF2 and CUL4A expression in colorectal cancer. Mol. Ther. Nucleic Acids *12*, 684–697.
11. Wang, X., Guo, B., Li, Q., Peng, J., Yang, Z., Wang, A., Li, D., Hou, Z., Lv, K., Kan, G., et al. (2013). miR-214 targets ATF4 to inhibit bone formation. Nat. Med. *19*, 93–100.
12. Zhang, K., Han, X., Zhang, Z., Zheng, L., Hu, Z., Yao, Q., Cui, H., Shu, G., Si, M., Li, C., et al. (2017). The liver-enriched lnc-LFAR1 promotes liver fibrosis by activating TGFβ and Notch pathways. Nat. Commun. *8*, 144.
13. Shi, Z., Chen, T., Yao, Q., Zheng, L., Zhang, Z., Wang, J., Hu, Z., Cui, H., Han, Y., Han, X., et al. (2017). The circular RNA ciRS-7 promotes APP and BACE1 degradation in an NF-κB-dependent manner. FEBS J. *284*, 1096–1109.
14. Feng, L., Shi, L., Lu, Y.F., Wang, B., Tang, T., Fu, W.M., He, W., Li, G., and Zhang, J.F. (2018). Linc-ROR Promotes Osteogenic Differentiation of Mesenchymal Stem Cells by Functioning as a Competing Endogenous RNA for miR-138 and miR-145. Mol. Ther. Nucleic Acids *11*, 345–353.
15. Li, C.J., Cheng, P., Liang, M.K., Chen, Y.S., Lu, Q., Wang, J.Y., Xia, Z.Y., Zhou, H.D., Cao, X., Xie, H., et al. (2015). MicroRNA-188 regulates age-related switch between osteoblast and adipocyte differentiation. J. Clin. Invest. *125*, 1509–1522.
16. Liao, J., Yu, X., Hu, X., Fan, J., Wang, J., Zhang, Z., Zhao, C., Zeng, Z., Shu, Y., Zhang, R., et al. (2017). lncRNA H19 mediates BMP9-induced osteogenic differentiation of mesenchymal stem cells (MSCs) through Notch signaling. Oncotarget *8*, 53581–53601.
17. Li, Z., Jin, C., Chen, S., Zheng, Y., Huang, Y., Jia, L., Ge, W., and Zhou, Y. (2017). Long non-coding RNA MEG3 inhibits adipogenesis and promotes osteogenesis of human adipose-derived mesenchymal stem cells via miR-140-5p. Mol. Cell. Biochem. *433*, 51–60.
18. Fabris, V.T. (2014). From chromosomal abnormalities to the identification of target genes in mouse models of breast cancer. Cancer Genet. *207*, 233–246.
19. Oury, F., Sumara, G., Sumara, O., Ferron, M., Chang, H., Smith, C.E., Hermo, L., Suarez, S., Roth, B.L., Ducy, P., and Karsenty, G. (2011). Endocrine regulation of male fertility by the skeleton. Cell *144*, 796–809.
20. Li, D.H., He, C.R., Liu, F.P., Li, J., Gao, J.W., Li, Y., and Xu, W.D. (2016). Annexin A2, up-regulated by IL-6, promotes the ossification of ligament fibroblasts from ankylosing spondylitis patients. Biomed. Pharmacother. *84*, 674–679.
21. Mukherjee, A., Larson, E.A., Klein, R.F., and Rotwein, P. (2014). Distinct actions of akt1 on skeletal architecture and function. PLoS ONE *9*, e93040.
22. Espirito Santo, A.I., Ersek, A., Freidin, A., Feldmann, M., Stoop, A.A., and Horwood, N.J. (2015). Selective inhibition of TNFR1 reduces osteoclast numbers and is differentiated from anti-TNF in a LPS-driven model of inflammatory bone loss. Biochem. Biophys. Res. Commun. *464*, 1145–1150.
23. Khurana, S., Buckley, S., Schouteden, S., Ekker, S., Petryk, A., Delforge, M., Zwijsen, A., and Verfaillie, C.M. (2013). A novel role of BMP4 in adult hematopoietic stem and progenitor cell homing via Smad independent regulation of integrin-α4 expression. Blood *121*, 781–790.
24. Evans, K.N., Potter, B.K., Brown, T.S., Davis, T.A., Elster, E.A., and Forsberg, J.A. (2014). Osteogenic gene expression correlates with development of heterotopic ossification in war wounds. Clin. Orthop. Relat. Res. *472*, 396–404.
25. Vanhaesebroeck, B., Guillermet-Guibert, J., Graupera, M., and Bilanges, B. (2010). The emerging mechanisms of isoform-specific PI3K signalling. Nat. Rev. Mol. Cell Biol. *11*, 329–341.
26. Kalinichenko, S.G., Matveeva, N.Y., Kostiv, R.E., and Puz', A.V. (2017). Role of Vascular Endothelial Growth Factor and Transforming Growth Factor-β2 in Rat Bone Tissue after Bone Fracture and Placement of Titanium Implants with Bioactive Bioresorbable Coatings. Bull. Exp. Biol. Med. *162*, 671–675.
27. Jin, C., Zheng, Y., Huang, Y., Liu, Y., Jia, L., and Zhou, Y. (2017). Long non-coding RNA MIAT knockdown promotes osteogenic differentiation of human adipose-derived stem cells. Cell Biol. Int. *41*, 33–41.
28. Bhushan, R., Grünhagen, J., Becker, J., Robinson, P.N., Ott, C.E., and Knaus, P. (2013). miR-181a promotes osteoblastic differentiation through repression of TGF-β signaling molecules. Int. J. Biochem. Cell Biol. *45*, 696–705.
29. Grey, A., Xu, X., Hill, B., Watson, M., Callon, K., Reid, I.R., and Cornish, J. (2004). Osteoblastic cells express phospholipid receptors and phosphatases and proliferate in response to sphingosine-1-phosphate. Calcif. Tissue Int. *74*, 542–550.
30. Teramachi, J., Hiruma, Y., Ishizuka, S., Ishizuka, H., Brown, J.P., Michou, L., Cao, H., Galson, D.L., Subler, M.A., Zhou, H., et al. (2013). Role of ATF7-TAF12 interactions in the vitamin D response hypersensitivity of osteoclast precursors in Paget's disease. J. Bone Miner. Res. *28*, 1489–1500.
31. Mahmood, A., Harkness, L., Schroder, H.D., Abdallah, B.M., and Kassem, M. (2010). Enhanced differentiation of human embryonic stem cells to mesenchymal progenitors by inhibition of TGF-beta/activin/nodal signaling using SB-431542. J. Bone Miner. Res. *25*, 1216–1233.
32. Pyott, S.M., Tran, T.T., Leistritz, D.F., Pepin, M.G., Mendelsohn, N.J., Temme, R.T., Fernandez, B.A., Elsayed, S.M., Elsobky, E., Verma, I., et al. (2013). WNT1 mutations in families affected by moderately severe and progressive recessive osteogenesis imperfecta. Am. J. Hum. Genet. *92*, 590–597.
33. Barnes, A.M., Carter, E.M., Cabral, W.A., Weis, M., Chang, W., Makareeva, E., Leikin, S., Rotimi, C.N., Eyre, D.R., Raggio, C.L., and Marini, J.C. (2010). Lack of cyclophilin B in osteogenesis imperfecta with normal collagen folding. N. Engl. J. Med. *362*, 521–528.
34. Tiaden, A.N., Breiden, M., Mirsaidi, A., Weber, F.A., Bahrenberg, G., Glanz, S., Cinelli, P., Ehrmann, M., and Richards, P.J. (2012). Human serine protease HTRA1 positively regulates osteogenesis of human bone marrow-derived mesenchymal stem cells and mineralization of differentiating bone-forming cells through the modulation of extracellular matrix protein. Stem Cells *30*, 2271–2282.
35. Nakajima, M., Takahashi, A., Tsuji, T., Karasugi, T., Baba, H., Uchida, K., Kawabata, S., Okawa, A., Shindo, S., Takeuchi, K., et al.; Genetic Study Group of Investigation Committee on Ossification of the Spinal Ligaments (2014). A genome-wide association study identifies susceptibility loci for ossification of the posterior longitudinal ligament of the spine. Nat. Genet. *46*, 1012–1016.

36. Byun, M.R., Hwang, J.H., Kim, A.R., Kim, K.M., Hwang, E.S., Yaffe, M.B., and Hong, J.H. (2014). Canonical Wnt signalling activates TAZ through PP1A during osteogenic differentiation. Cell Death Differ. *21*, 854–863.

37. Xiang, X., Zhao, J., Xu, G., Li, Y., and Zhang, W. (2011). mTOR and the differentiation of mesenchymal stem cells. Acta Biochim. Biophys. Sin. (Shanghai) *43*, 501–510.

38. Wang, N., Zhou, Z., Wu, T., Liu, W., Yin, P., Pan, C., and Yu, X. (2016). TNF-α-induced NF-κB activation upregulates microRNA-150-3p and inhibits osteogenesis of mesenchymal stem cells by targeting β-catenin. Open Biol. *6*, 150258.

39. Wang, Z., Xie, Q., Yu, Z., Zhou, H., Huang, Y., Bi, X., Wang, Y., Shi, W., Sun, H., Gu, P., and Fan, X. (2015). A regulatory loop containing miR-26a, GSK3β and C/EBPα regulates the osteogenesis of human adipose-derived mesenchymal stem cells. Sci. Rep. *5*, 15280.

40. Beermann, J., Piccoli, M.T., Viereck, J., and Thum, T. (2016). Non-coding RNAs in Development and Disease: Background, Mechanisms, and Therapeutic Approaches. Physiol. Rev. *96*, 1297–1325.

41. Kong, P., Zhu, X., Geng, Q., Xia, L., Sun, X., Chen, Y., Li, W., Zhou, Z., Zhan, Y., and Xu, D. (2017). The microRNA-423-3p-Bim Axis Promotes Cancer Progression and Activates Oncogenic Autophagy in Gastric Cancer. Mol. Ther. *25*, 1027–1037.

42. Ning, S., Chen, Z., Fan, D., Sun, C., Zhang, C., Zeng, Y., Li, W., Hou, X., Qu, X., Ma, Y., and Yu, H. (2017). Genetic differences in osteogenic differentiation potency in the thoracic ossification of the ligamentum flavum under cyclic mechanical stress. Int. J. Mol. Med. *39*, 135–143.

43. Qu, X., Chen, Z., Fan, D., Sun, C., and Zeng, Y. (2016). MiR-132-3p Regulates the Osteogenic Differentiation of Thoracic Ligamentum Flavum Cells by Inhibiting Multiple Osteogenesis-Related Genes. Int. J. Mol. Sci. *17*, 1370.

44. Zhang, J., Li, Z., Liu, L., Wang, Q., Li, S., Chen, D., Hu, Z., Yu, T., Ding, J., Li, J., et al. (2017). Long Noncoding RNA TSLNC8 Is a Tumor Suppressor that Inactivates the IL-6/STAT3 Signaling Pathway. Hepatology *67*, 171–187.

45. Li, F., Zhang, L., Li, W., Deng, J., Zheng, J., An, M., Lu, J., and Zhou, Y. (2015). Circular RNA ITCH has inhibitory effect on ESCC by suppressing the Wnt/β-catenin pathway. Oncotarget *6*, 6001–6013.

46. Hansen, T.B., Jensen, T.I., Clausen, B.H., Bramsen, J.B., Finsen, B., Damgaard, C.K., and Kjems, J. (2013). Natural RNA circles function as efficient microRNA sponges. Nature *495*, 384–388.

47. Memczak, S., Jens, M., Elefsinioti, A., Torti, F., Krueger, J., Rybak, A., Maier, L., Mackowiak, S.D., Gregersen, L.H., Munschauer, M., et al. (2013). Circular RNAs are a large class of animal RNAs with regulatory potency. Nature *495*, 333–338.

OMTN, Volume *12*

Supplemental Information

A Transcriptome-Level Study Identifies Changing Expression Profiles for Ossification of the *Ligamentum Flavum* of the Spine

Yawei Han, Yuheng Hong, Liandong Li, Tengshuai Li, Zhen Zhang, Jingzhao Wang, Han Xia, Yutao Tang, Zhemin Shi, Xiaohui Han, Ting Chen, Qi Liu, Mengxia Zhang, Kun Zhang, Wei Hong, and Yuan Xue

Supplementary Figures

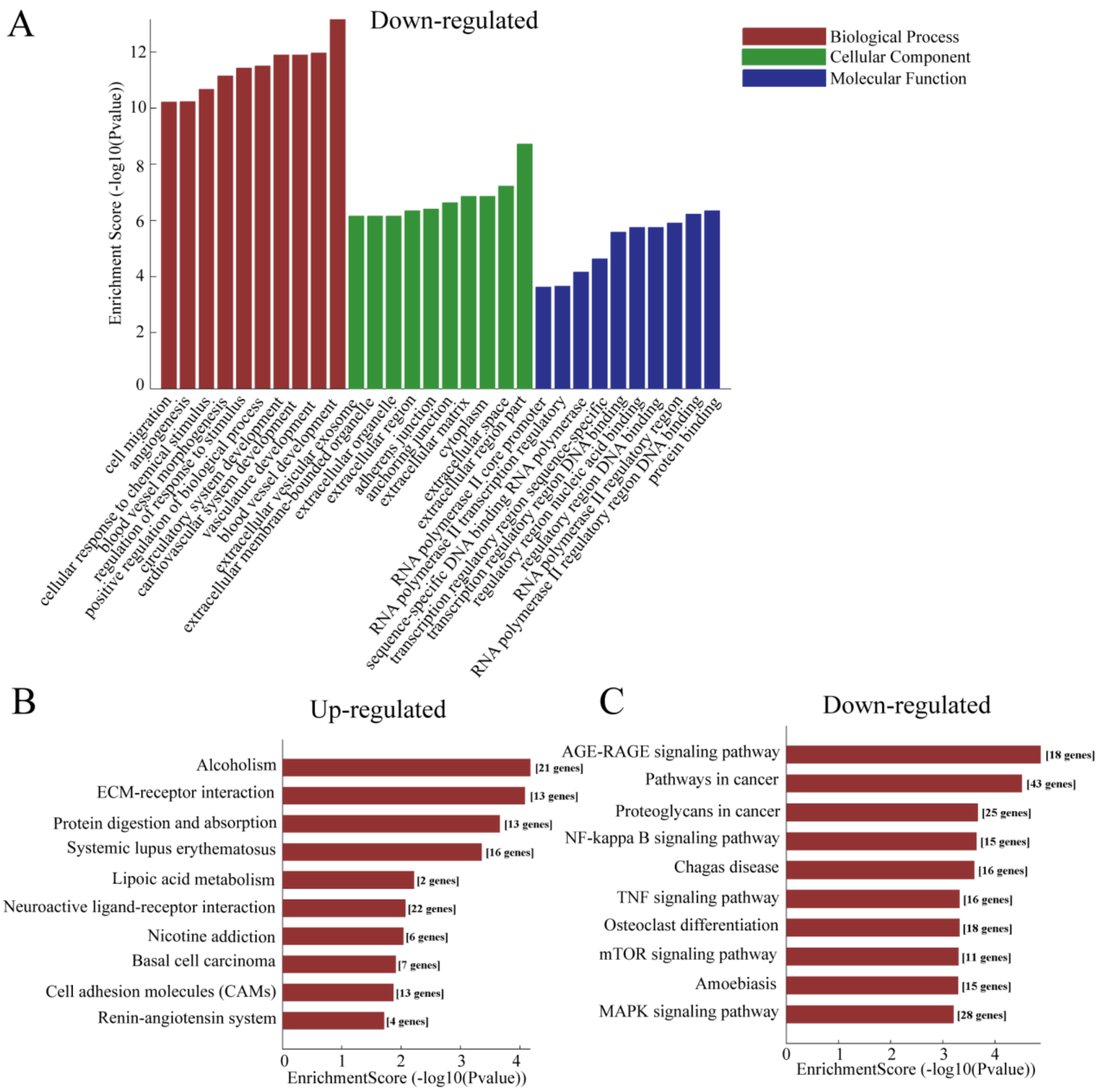

Supplementary Figure 1. GO and KEGG pathway analysis of differentially expressed mRNAs. (A) GO annotation of the down-regulated mRNAs with top ten enrichment score covering domains of biological processes, cellular components and molecular functions were performed. (B-C) KEGG pathway enrichment analysis of up- (B) and down-regulated (C) mRNAs with top ten enrichment score was performed. Both up- and down-regulated mRNAs are significantly changed with fold change > 2.0, $p < 0.05$ between OLF and normal LF tissues.

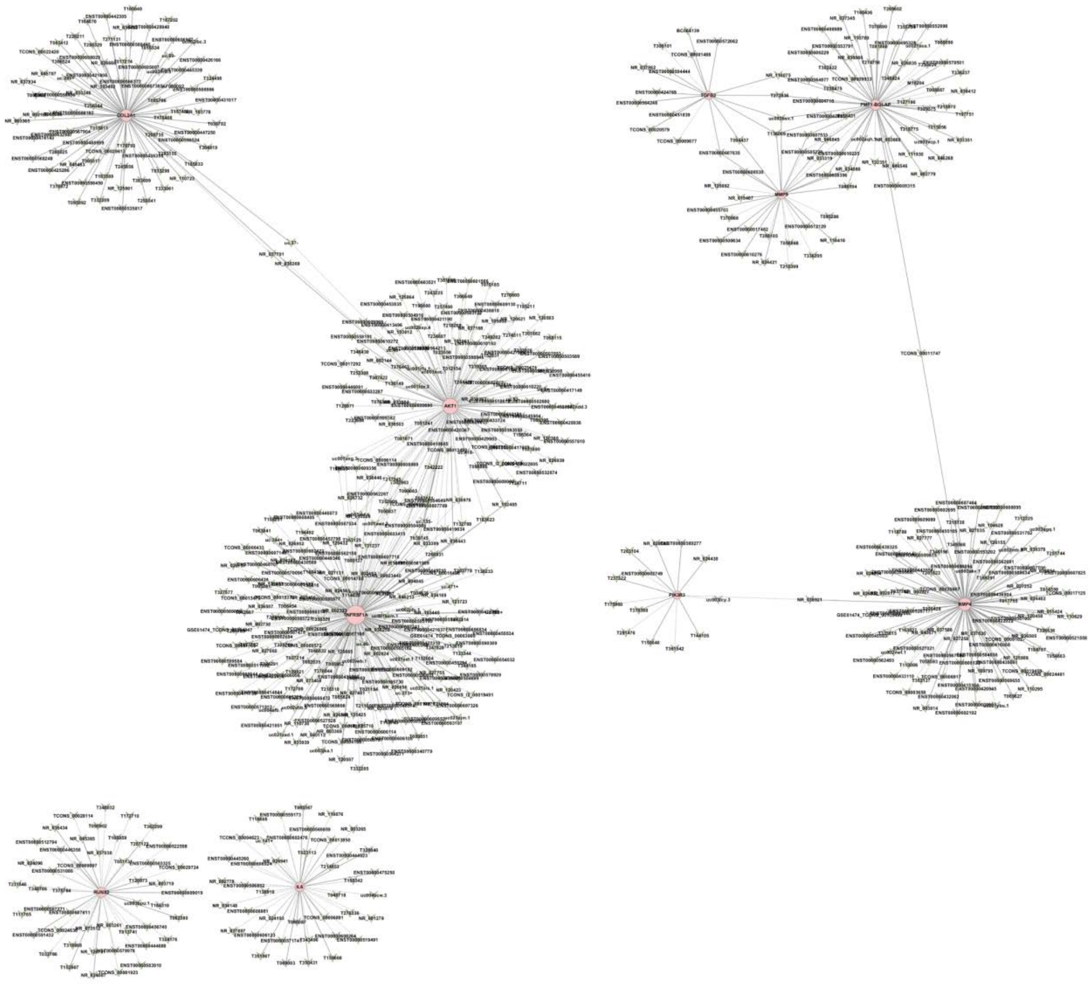

Supplementary Figure 2. Co-expression network of ten significantly deregulated mRNAs and their co-expressed lncRNAs. The coding genes, including RUNX2, MMP9, BGLAP, IL6, COL2α1, AKT1, TNFRSF1A, TGFB2, BMP4 and PIK3R3, and their co-expressed lncRNAs, that have Pearson correlation coefficients≥0.968 were chosen to draw the network using Cytoscape. V nodes represent lncRNA and circle nodes represent mRNAs. Solid lines represent positive relationship and dash lines represent negative relationship.

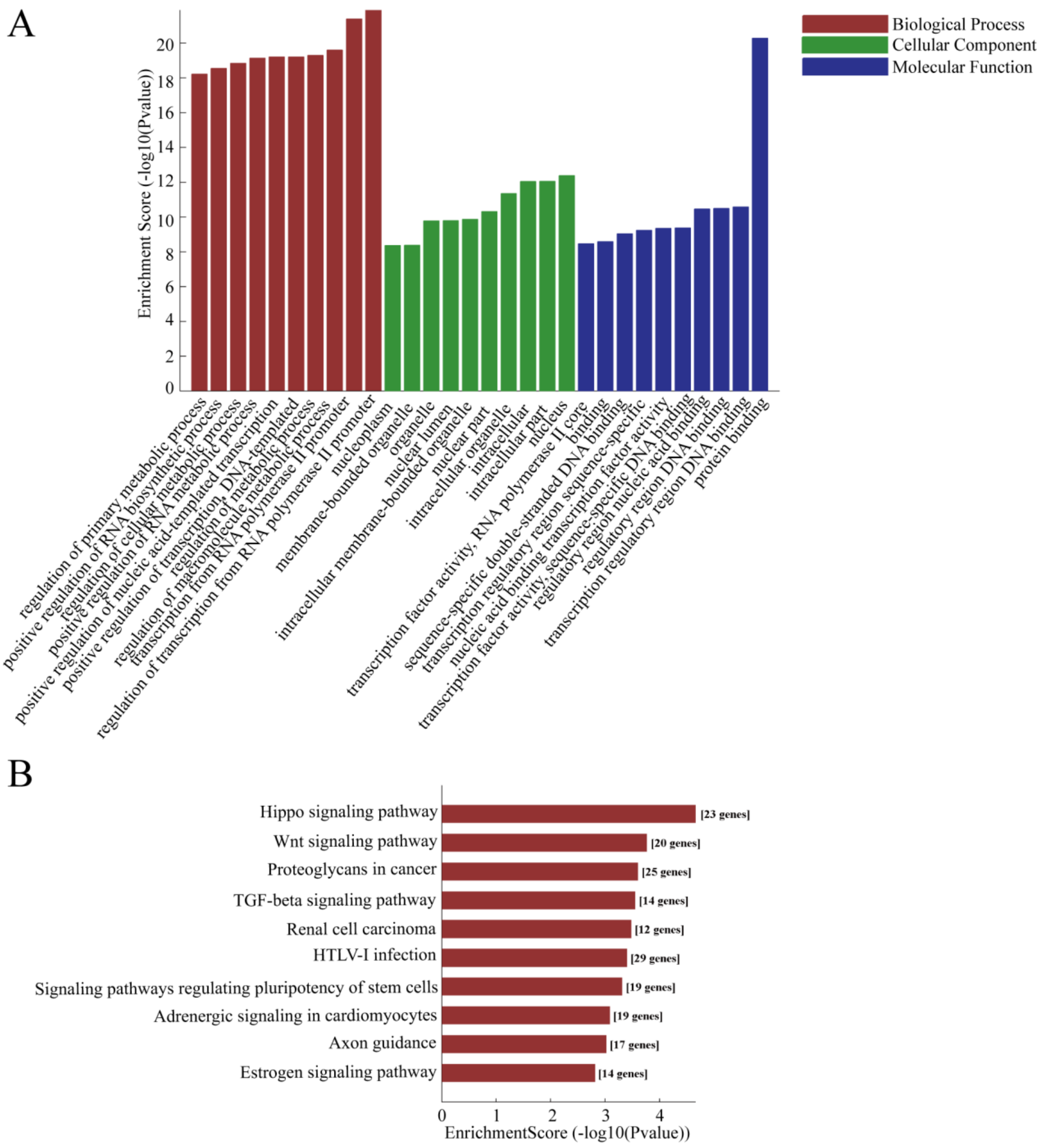

Supplementary Figure 3. GO and KEGG pathway analysis of miRNAs target genes. (A) GO annotation of all targeted mRNAs of deregulated miRNAs with top ten enrichment score covering domains of biological processes, cellular components and molecular functions was performed. (B) KEGG pathway enrichment analysis of targeted mRNAs of all deregulated miRNA with top ten enrichment score was performed. Both up- and down-regulated miRNAs are significantly changed with fold change>2.0, $p<0.05$ between OLF and normal LF tissues.

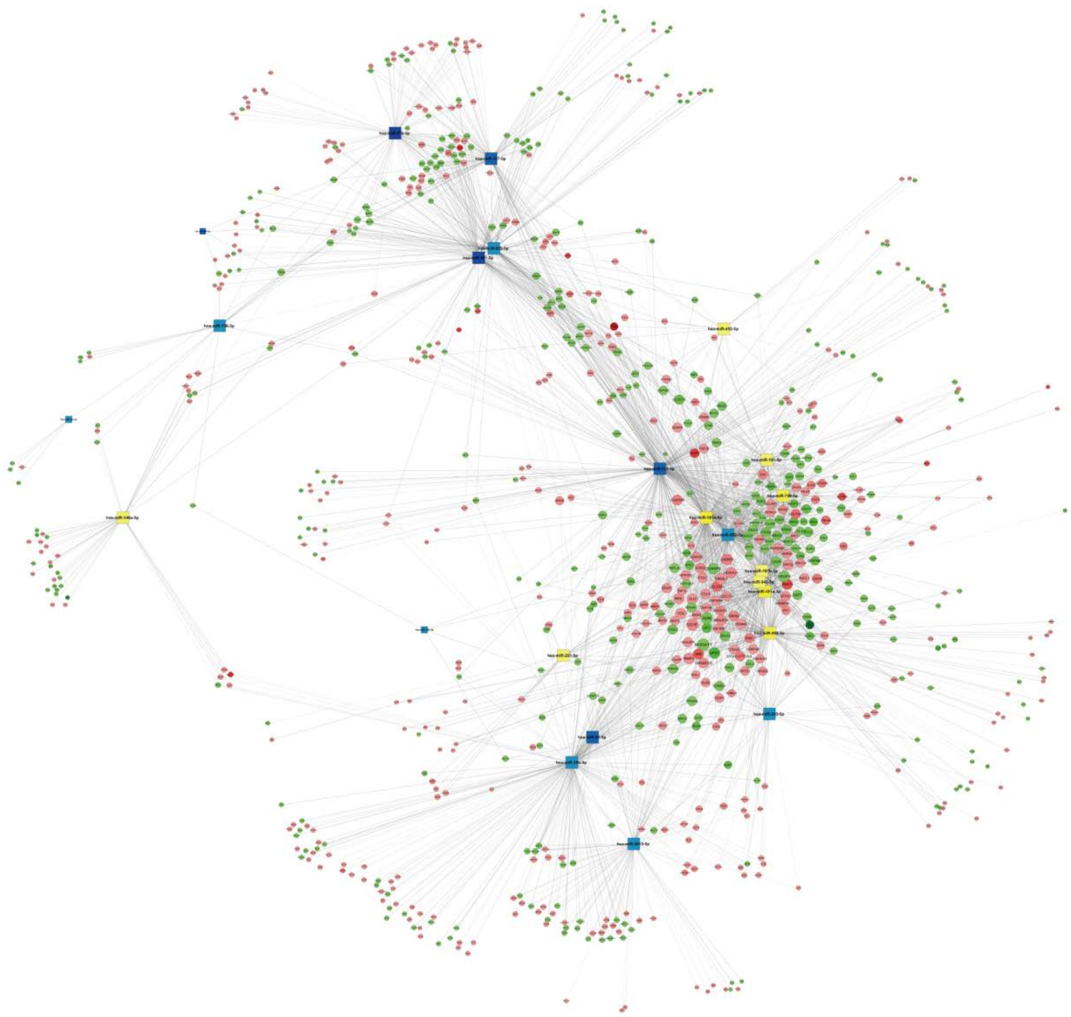

Supplementary Figure 4. The miRNA-mRNA interacting network. The differentially expressed mRNAs identified previously (Figure 1A) overlap with the predicted miRNAs targets, and only the genes that negatively correlated with its corresponding miRNAs are included. These inversely correlated miRNA/mRNA pairs were sorted to construct the networks. Circle nodes represent mRNAs and square nodes represent miRNAs. Red and yellow color represent up regulation, and green and blue color represent down regulation respectively. The shade darkness represents fold change. The size of circle represents *p* value with larger size owing smaller *p* value. Solid lines represent positive relationship and dash lines represent negative relationship.

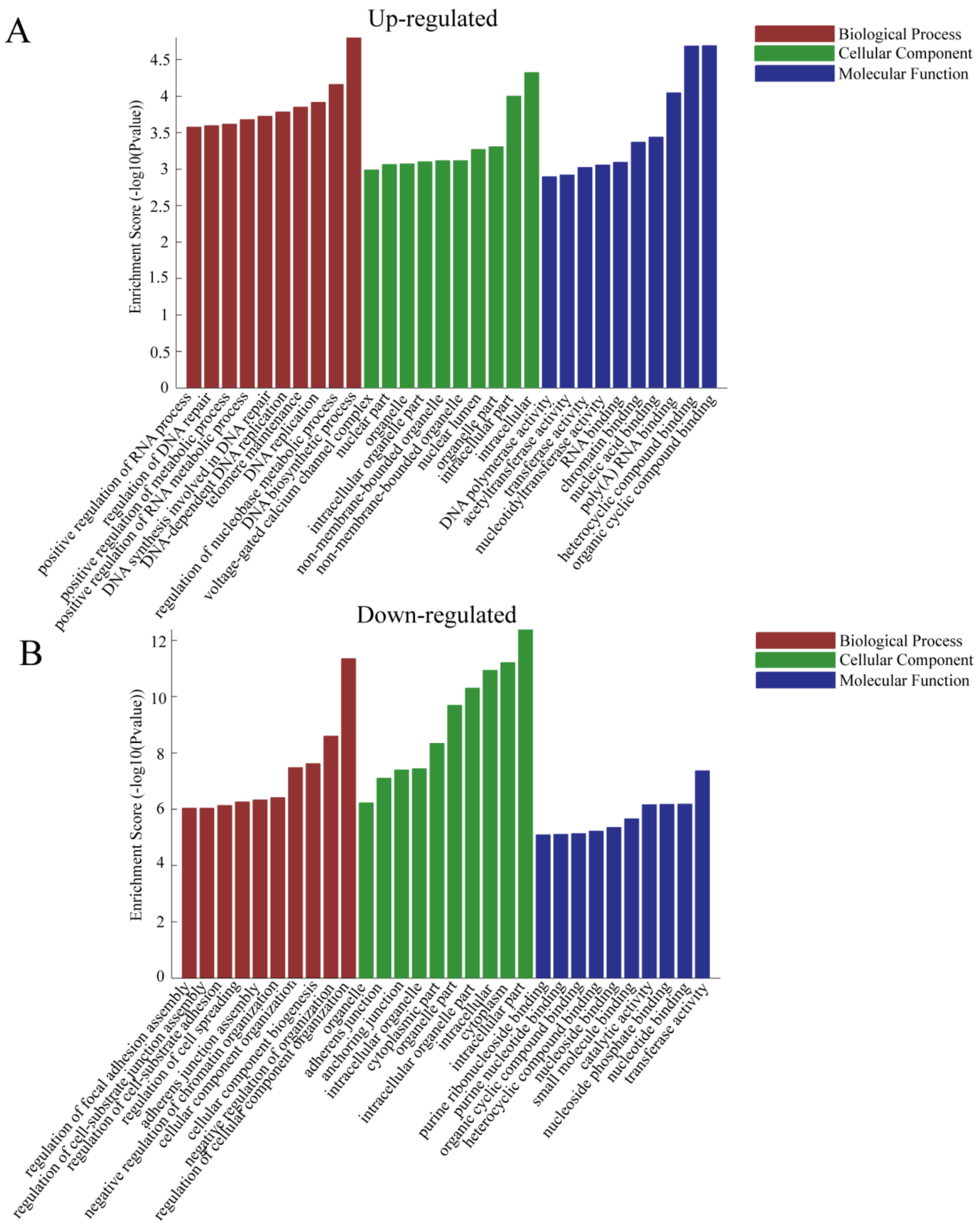

Supplementary Figure 5. GO and KEGG pathway analysis of circRNAs-related linear counterparts. (A-B) GO annotation of the linear counterparts of up- (A) and down-regulated (B) circRNAs with top ten enrichment score covering domains of biological processes, cellular components and molecular functions was performed. Both up- and down-regulated miRNAs are significantly changed with fold change > 2.0, $p < 0.05$ between OLF and normal LF tissues.

Supplementary Table 1. The information about the patients

Serial number	Age	Sex	Disease	Tissue
OLF-1#	56	F	Spinal stenosis	Ossified ligamentum flavum
OLF-2#	57	M	Spinal stenosis	Ossified ligamentum flavum
OLF-3#	60	M	Spinal stenosis	Ossified ligamentum flavum
OLF-4#	68	M	Spinal stenosis	Ossified ligamentum flavum
LF-1#	42	M	Spinal trauma	Normal ligamentum flavum
LF-2#	57	M	Spinal trauma	Normal ligamentum flavum
LF-3#	73	M	Spinal trauma	Normal ligamentum flavum
LF-4#	40	F	Spinal trauma	Normal ligamentum flavum

Supplementary Table 2. The deregulated expression of mRNAs in microarray in OLF

Gene Symbol	Seqname	Regulation	Fold Change	*p* value
AK5	NM_174858	up	24.6237614	0.002139029
COL2A1	NM_001844	up	24.3281001	0.000183825
PMF1-BGLAP	NM_001199661	up	14.0520984	0.001964946
RUNX2	NM_001024630	up	10.5248663	0.04779347
BMP4	NM_001202	up	4.7992443	0.00207799
IL6	NM_000600	down	62.2617382	0.027100353
CRACR2B	NM_001286606	down	20.0370609	0.018754584
SAA1	NM_000331	down	14.8008962	0.00492719
G0S2	NM_015714	down	13.6180691	0.007933331
ATF3	NM_001674	down	7.0536263	0.002535643

Supplementary Table 3. The deregulated expression of lncRNAs in microarray in OLF

Gene Symbol	Seqname	Regulation	Fold Change	*p* value
RP11-644F5.16	ENST00000556606	up	48.3224434	0.002456396
LOC101927269	NR_110075	up	31.1198732	0.001515081
LINC00347	NR_034024	up	17.0523229	0.001624259
CTD-2249K22.1	ENST00000512859	up	2.275214	0.013428992
LINC01135	NR_034015	up	2.9781847	0.000480586
LOC100505817	NR_038340	down	25.5974901	0.027545948
RP11-109E24.1	ENST00000511279	down	11.7276114	0.004813486
AQP7P3	NR_026558	down	9.1849429	0.010875482
RP11-720L8.1	ENST00000561054	down	6.9156078	0.001576764
lncMIAT	NR_033319	down	2.6070678	0.015431081
RP11-523G9.3	ENST00000608133	up	2.5794996	0.029309278
CTB-180A7.6	ENST00000599584	up	2.5120166	0.013285599

Supplementary Table 4. The deregulated expression of miRNAs in sequencing in OLF

Gene Symbol	Regulation	Fold Change	*p* value
hsa-miR-181a-5p	up	5.013659	9.87557E-05
hsa-miR-181a-3p	up	4.609562	0.004157204
hsa-miR-708-5p	up	3.632302	0.009366564
hsa-miR-181b-5p	up	2.738144	0.006102139
hsa-miR-146a-5p	up	2.538066	0.023043047
hsa-miR-889-3p	down	2.949365297	0.007410704
hsa-miR-32-5p	down	3.371430498	0.03504732
hsa-miR-379-5p	down	4.340579207	0.02562263
hsa-miR-novel-chrX_47539	down	5.016881807	0.007573798
hsa-miR-381-3p	down	5.930389093	6.51417E-05
hsa-miR-19b-3p	down	2.069497878	0.006346186

Supplementary Table 5. The deregulated expression of circRNAs in microarray

Gene Symbol	Regulation	Fold Change	*p* value
hsa_circ_0000367	up	30.7576643	2.25293E-06
hsa_circ_0004183	up	20.0548723	0.002363038
hsa_circ_0006220	up	16.0456877	3.82059E-06
hsa_circ_0001666	up	37.4327367	4.4194E-06
hsa_circ_0004099	up	28.1184624	0.002744821
hsa_circ_0001873	down	4.6512421	0.000257104
hsa_circ_0023016	down	15.5507265	0.028144848
hsa_circ_0003748	down	15.0729102	0.021007797
hsa_circ_0092360	down	14.1954431	0.036578546
hsa_circ_0001296	down	13.4871247	0.016418551
hsa_circ_0050139	up	2.3049384	0.003656394

Supplementary Table 6. Primers for qPCR, Related to Experimental Procedures

qPCR primers for analysis of mRNA

Gene symbol	Forward 5' - 3'	Reverse 5' - 3'
AK5	CCTGATGAAGAGGGCATTGT	TGATTAGCACAAGCCAGGAA
COL2A1	AAGGGTGATCGTGGTGAGAC	GCACCAGCTTCTCCTCTGTC
CRACR2B	ACAAGGAGGCTAAGGGCTTC	GGTGAGGAAGCCAGTGTGA
Runx2	TGCGGCCGCCCCACGACAA	ACCCGCCATGACAGTAACCACAGT
BGLAP	CACTCCTCGCCCTATTGGC	CCCTCCTGCTTGGACACAAAG
SAA1	CGGCTCAGACAAATACTTCCA	CACCATGGCCAAAGAATCTC
IL6	CTCAATATTAGAGTCTCAACCCCCA	GAGAAGGCAACTGGACCGAA
BMP4	CGTCCAAGCTATCTCGAGCC	CATAGGTCCCTGCAGTAGCG
G0S2	GCCGTGCCACTAAGGTCATT	GATCAGCTCCTGGACCGTTT
ATF3	TGATGCTTCAACACCCAGGC	GGATGGCAAACCTCAGCTCT
GAPDH	ACCCAGAAGACTGTGGATGG	TTCAGCTCAGGGATGACCTT

qPCR primers for analysis of lncRNA

Seqname	Forward 5' - 3'	Reverse 5 '- 3'
ENST00000556606	AGAGGCTGGGCTACAGAAGAA	GAGGCTCTGCTCTTGTGCG
ENST00000512859	GTGTGGGGTACGTTCACCTT	ACGGGCAGGTTTATTTGGGT
ENST00000511279	TGCCAGATTCATGATGCCAC	GCTCCTCAGTTGTAGCCTCTC
ENST00000561054	TGGGGAACCAGCAAACTCAT	ACTGACAAACTCCCACGCTG
NR_033319	AGGATTCCTTGCATGGGGTG	CCCAAGAAGCACCGTGAGAT
NR_110075	GAGCTGTAGCAGAAAAGCAAGT	TCCAATCCATTGTCCACGCT
NR_034024	GAGGAGGGGCCTTTCATAGC	CAATTACCACAGGCGGACCT
NR_034015	AGCGCTAGACACATGGAACC	TTCCAGCGCGACTGTTGTTA
NR_038340	GGTGACCTGGGCTCCTTATG	CTGCAAGTGTCTTCCTGTGGA
NR_026558	CATGGTTCAAGCATCTGGGC	TAGAGCCTGCATCGTGGTTC
ENST00000608133	AGAGGCATTTGTCGGTAGTTTT	TCTGATGGGCATCCACACATT
ENST00000599584	CCGAGGAGGGTTTCTCTAGC	GTCCCTCCACAGCCCTTAAC

qPCR primers for analysis of miRNA

Gene symbol	primers
hsa-miR-181a-5p	AACATTCAACGCTGTCGGTGA
hsa-miR-181a-3p	ACCATCGACCGTTGATTGTAC
hsa-miR-708-5p	AAGGAGCTTACAATCTAGCTGG
hsa-miR-181b-5p	AACATTCATTGCTGTCGGTGG
hsa-miR-146a-5p	ATGAGAACTGAATTCCATGGGTT
hsa-miR-379-5p	TGGTAGACTATGGAACGTAGG
hsa-miR-novel-chrX_47539	ACTCGATGTTGGATCAGGACA
hsa-miR-381-3p	TATACAAGGGCAAGCTCTCTGT
hsa-miR-889-3p	CGTTAATATCGGACAACCATTGT
hsa-miR-32-5p	CGTATTGCACATTACTAAGTTGCA
hsa-miR-19b-3p	TGTGCAAATCCATGCAAAACTGA

qPCR primers for analysis of circRNA

Gene symbol	Forward 5' - 3'	Reverse 5 '- 3'
hsa_circ_0000367	AGACTGGCGTGAAACCTTCC	CGTATGGAATGGACCTGGACA
hsa_circ_0001873	CTGAGCTCATGTGAGGGTGA	TCGTGCGGATGATCGGGATA
hsa_circ_0004183	CGTCCATTCCACGAGGTTCT	TTCAAGGCAGCCAGCATGTC
hsa_circ_0006220	TGCATCTACCCTGCTGAACCT	TTGGCTACATCCTGCAGTGAAA
hsa_circ_0023016	GAGAGTCTCACTGCTCCAGGG	CCACACACATCCCACTACCC
hsa_circ_0003748	TGTGGCATGCAGCCATAAGTT	GCTGGTGACATTTCATGCTC
hsa_circ_0001666	GCCTAGCTGTCAAGGAGTGG	CTTGCAAACCTCTCACACCCA
hsa_circ_0004099	AGGTTTTTGAACTGCTCGGGG	GGCACCATCCCTGGCTTTAGA
hsa_circ_0092360	GGCCGGAAGTGACCGATTTC	CAGCCGTGTTCCCAATTCGT
hsa_circ_0001296	GTGTGCTGTGATGAGGAAGATGA	AGCACGCCGTTCAATCACAT
hsa_circ_0050139	CCCCATGACTCAGGGTTTTGA	TCTTCTTGCGGCAGTTGACA

siRNA sequences for lncRNAs and circRNA

Gene symbol	Forward 5' - 3'	Reverse 5 '- 3'
siENST00000608133	GAAGGCACCAUUUAACAAATT	UUUGUUAAAUGGUGCCUUCTT
siENST00000599584	GGCUUUGGAUGAGACCGUATT	UACGGUCUCAUCCAAAGCCTT
sihsa_circ_0050139	GCUGCUCAGUGCUAUGCUCTT	GAGCAUAGCACUGAGCAGCTT
